# Autoimmune diseases and risk of adverse pregnancy outcomes: a population-based cohort study of five million pregnancies in the UK

**DOI:** 10.1186/s12916-026-04921-w

**Published:** 2026-05-21

**Authors:** Megha Singh, Anuradhaa Subramanian, Steven Wambua, Neil Cockburn, Stephanie J Hanley, Siang Ing Lee, Arturo Gonzalez-Izquierdo, Mairead Black, John A Reynolds, Francesca L Crowe, Krishnarajah Nirantharakumar

**Affiliations:** 1 Department of Applied Health Sciences, University of Birmingham, Birmingham, UK; 2 Aberdeen Centre for Women’s Health Research, University of Aberdeen, Aberdeen, Aberdeenshire, UK; 3 Department of Inflammation and Ageing, University of Birmingham, Birmingham, UK; 4 School of Life Course and Population Sciences, King’s College London, London, UK

**Keywords:** Adverse pregnancy outcomes, Autoimmune disease, Caesarean birth, Clinical Practice Research Datalink, Gestational hypertension, Mental health, Miscarriage, Preterm birth, Stillbirth, Type 1 diabetes, systemic lupus erythematosus

## Abstract

**Background:**

With an increasing trend in the prevalence of maternal autoimmune diseases in pregnancy, there is need for evidence on the association between autoimmune diseases and pregnancy outcomes.

**Methods:**

This population-based cohort study used data on pregnancies from primary care practices that contributed to the UK Clinical Practice Research Datalink (CPRD) database (Gold and Aurum) between 2000 and 2022, linked to Hospital Episode Statistics (HES). Modified Poisson regression with robust standard errors estimated adjusted relative risks (aRR) and 95% confidence intervals (95% CI) assessing the association between 17 autoimmune diseases and 12 pregnancy outcomes, selected following literature review and expert consultation. Models were adjusted for demographic and comorbidity variables. Findings were evaluated using the Benjamini–Yekutieli procedure to control for multiple testing across autoimmune disease–outcome associations.

**Results:**

A total of 5,239,383 pregnancies from the CPRD pregnancy register and 2,485,366 births recorded in HES maternity data met the eligibility criteria. Women with autoimmune diseases had an increased risk across all pregnancy outcomes examined. Among antenatal outcomes, markedly elevated risks were observed for hyperemesis gravidarum in Addison’s disease (aRR 3.72, 95% CI 2.48–5.59), miscarriage in Sjögren’s syndrome (1.66, 1.02–2.70), gestational hypertension in type 1 diabetes mellitus (T1DM; 2.95, 2.77–3.15), pre-eclampsia/eclampsia in T1DM (3.56, 3.32–3.82), and gestational diabetes mellitus in Graves’ disease (1.37, 1.21–1.54).

For obstetric outcomes, increased risks were observed for caesarean birth in inflammatory bowel disease (IBD; 1.27, 1.22–1.31), small for gestational age in systemic lupus erythematosus (SLE; 2.45, 1.65–3.62), preterm birth in rheumatoid arthritis (1.53, 1.33–1.76), and stillbirth in SLE (1.82, 1.12–1.84).

Perinatal mental health outcomes were more common across several autoimmune diseases, with particularly high risks in myasthenia gravis (anxiety 3.05, 1.89–4.92; depression 1.77, 1.37–2.28), SLE (anxiety 2.11, 1.67–2.67; depression 1.36, 1.22–1.53), and multiple sclerosis (anxiety 1.76, 1.38–2.23; depression 1.94, 1.77–2.12). Inverse associations were observed for hyperemesis gravidarum in systemic sclerosis, SLE, and myasthenia gravis, and for hypertensive disorders of pregnancy in multiple sclerosis. After Benjamini–Yekutieli correction for multiple testing, the number of statistically significant associations was reduced, with a core set of robust associations persisting across selected autoimmune diseases particularly, T1DM, SLE, Graves’ disease, IBD and key pregnancy outcomes.

**Conclusions:**

Autoimmune diseases were associated with increased risks across a wide range of adverse pregnancy outcomes, with marked heterogeneity between individual conditions. This study provides adjusted relative risks across multiple domains of pregnancy outcomes including antenatal (e.g. miscarriage, hyperemesis gravidarum, gestational hypertension, pre-eclampsia, gestational diabetes), obstetric (e.g. preterm birth, caesarean birth, small for gestational age, stillbirth), and perinatal mental health outcomes (anxiety and depression), for both common and less frequently studied autoimmune diseases. The findings highlight the importance of disease-specific evaluation of pregnancy risks.

**Supplementary Information:**

The online version contains supplementary material available at 10.1186/s12916-026-04921-w.

## Background

Autoimmune diseases encompass a diverse range of conditions, typically associated with autoantibody development, chronic inflammation, and tissue damage [1]. Common autoimmune conditions include type 1 diabetes mellitus (T1DM), rheumatoid arthritis, multiple sclerosis, and systemic lupus erythematosus (SLE) [2, 3]. Most autoimmune diseases disproportionately affect women with a higher incidence compared to men although the female to male ratio varies between conditions. For instance, when compared to men, women are nine times more likely to develop SLE and three times more likely to develop rheumatoid arthritis [4]. The increasing trend in autoimmune diseases among women and hence in pregnancy [5] has been attributed to a combination of factors including genetic susceptibility, hormonal influences, and environmental factors, underscoring the need for a greater understanding of their impact on women’s health [6, 7].

In an umbrella review, we investigated the relationship between autoimmune diseases and pregnancy complications [8]. For example, women with SLE had a higher risk of miscarriage and preterm birth [9–12]. Similarly, rheumatoid arthritis was associated with an increased incidence of gestational hypertension and pre-eclampsia [13, 14], and T1DM was linked to the development of anxiety and depression during pregnancy [15, 16]. In the UK context, a study using the Clinical Practice Research Datalink (CPRD) found that women with psoriasis or T1DM had higher rates of miscarriage or preterm birth and women with coeliac disease or inflammatory bowel disease (IBD) are at higher risk of postpartum haemorrhage compared to women without these health conditions [17–21]. Furthermore, women with autoimmune diseases had a 30% higher risk of developing a mental health condition [22, 23]. We identified an important knowledge gap in our understanding of pregnancy complications in women with less common autoimmune diseases such as myasthenia gravis or Addison’s disease and rarer outcomes in common autoimmune diseases.

This study aims to comprehensively analyse the association between a range of autoimmune diseases and key pregnancy complications, including hyperemesis gravidarum, ectopic pregnancy, miscarriage, gestational hypertension, pre-eclampsia/eclampsia (PE/eclampsia), gestational diabetes mellitus (GDM), preterm birth, and newly developed mental health conditions during pregnancy (anxiety or depression). Utilising the large and diverse UK CPRD database [24, 25], this research will offer novel insights into the specific risks posed by different autoimmune conditions during pregnancy. It is expected that the findings will enhance our understanding of how autoimmune diseases impact pregnancy, thereby informing clinical management practices to improve maternal and fetal health outcomes.

## Methods

### Ethical approval

for this study was approved by Independent Scientific Advisory Committee for research involving CPRD data (protocol no. 23_002650) [26, 27]. The study results are reported as per the STROBE (Strengthening the reporting of observational studies in epidemiology) statement (additional file 1 Table 1) [28] .

### Study design and data sources

This is a retrospective cohort study utilising data from the CPRD, a large UK-based primary care database. The CPRD contains anonymised patient-level data, including demographics, symptoms, diagnoses, drug prescriptions, physical measurements, and laboratory test results [24, 25]. The study combines data from two CPRD databases: CPRD Gold and CPRD Aurum, which are linked to general practices using VISION and OPTUM software, respectively. CPRD Gold includes records from 985 general practices, while CPRD Aurum covers 1,489 practices, representing approximately 5% and 20% of the UK population, respectively. Both CPRD Gold and CPRD Aurum maintain Pregnancy Registers derived from electronic health records (EHRs) in primary care, consolidating information on pregnancy dates, related covariates, and outcomes. For this study, maternal primary care records from these databases, along with their respective Pregnancy Registers, were used to gather data on exposure to pre-existing autoimmune diseases, relevant covariates before pregnancy, and pregnancy outcomes [29–31].

We utilised CPRD linked births recorded in the Hospital Episodes Statistics (HES) maternity records dataset to accurately capture obstetric outcomes. Additionally, secondary care outcomes were obtained using linked HES Admitted Patient Care data, which captures inpatient records from general National Health Service hospitals [24, 25, 29–31]. HES Admitted Patient Care data provides diagnostic records for secondary care inpatient admissions, but is only available for England, thus restricting the analysis to pregnancies within England. Data on exposure, outcomes and covariates were extracted using the data extraction for epidemiological research (DExtER) tool, which facilitates extraction based on predefined parameters [32].

### Study Population

There were two study cohorts based on the types of outcomes studied.

#### (i) Pregnancy cohort

This cohort included pregnant women aged 15 to 49 from the Pregnancy Register between 2000 to 2022. It was used to study antenatal outcomes and mental health outcomes, which were ascertained in both primary care and linked HES secondary care records. This cohort included pregnant women who were not eligible for HES linkage. Pregnancies were the unit of analysis.

#### (ii) Birth cohort

This cohort included pregnant women aged 15 to 49, who had a birth recorded in the HES maternity dataset between 2000 and 2022. It was used to study obstetric outcomes including preterm birth, small for gestational age (SGA), caesarean birth, and stillbirth. These outcomes were ascertained from HES records. Births were the unit of analysis. Women identified in the HES maternity records were linked back to primary care data to ascertain their autoimmune disease exposure status. This cohort is limited to England.

### Data quality

**Fig. 1:**
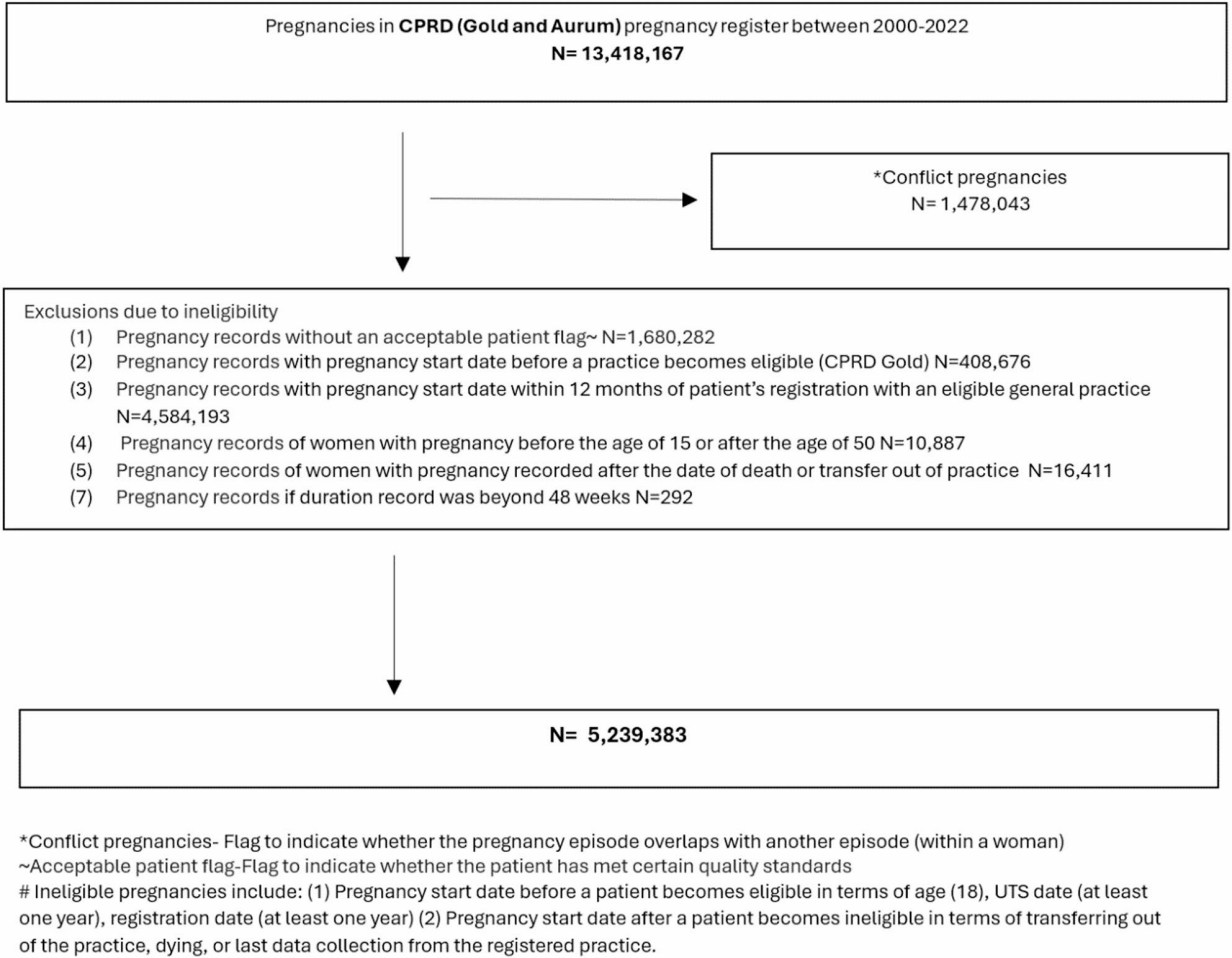
Flowchart describing pregnancy cohort selection

**Fig. 2:**
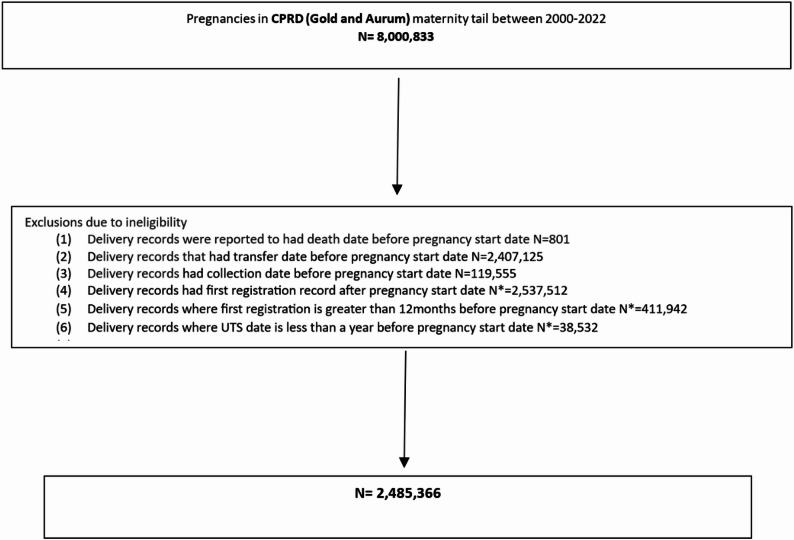
Flowchart describing birth cohort (HES maternity tail) selection. *Data quality check, UTS=Up to standard date

Pregnancies and births were the units of analysis, to capture key obstetric outcomes such as preterm birth, SGA, caesarean section, and stillbirth. To ensure data quality, pregnancies were included after the mother’s record received an acceptable patient flag from CPRD and after a minimum of one year of registration with a practice. All eligible women meeting these criteria were included in the study [24, 25]. The cohort selection criteria are detailed in Figures 1, 2, and additional file 2: figure 1-4. The comparator group comprised pregnancies/births among women with no recorded diagnosis of any autoimmune disease in CPRD or HES prior to or during pregnancy.

### Exposure Definition

Autoimmune diseases were identified using primary care records based on diagnostic codes (Read and SNOMED CT in CPRD Gold and Aurum, respectively) [33, 34]. The study encompassed 17 autoimmune conditions; (1) Addison’s disease, (2) alopecia areata, (3) Ankylosing spondylitis (4) coeliac disease, (5) inflammatory bowel diseases (IBD) including Crohn’s disease and ulcerative colitis, (6) Graves’ disease, (7) Hashimoto’s thyroiditis, (8) multiple sclerosis, (9) myasthenia gravis, (10) psoriasis, (11) psoriatic arthritis (12) rheumatoid arthritis, (13) Sjögren’s syndrome, (14) SLE, (15) systemic sclerosis, (16) T1DM and (17) vitiligo. Selection of these conditions was based on their prevalence in women of reproductive age, informed by literature review and expert consultation [35]. For the ease of description, these diseases are grouped according to the organ system namely systemic inflammatory disorders, dermatological disorders, endocrine disorders, gastrointestinal disorders, musculoskeletal disorders, and neurological disorders. If more than one autoimmune diagnosis was recorded for a woman in relation to the same pregnancy, the pregnancy was classified according to the first-ever recorded diagnosis (primary diagnosis). Diagnostic codes were identified from all available records prior to pregnancy start, rather than at recruitment, thereby capturing pre-existing autoimmune conditions. This ensured that autoimmune diseases were treated as pre-pregnancy exposures, and outcomes were categorised into antenatal (occurring during pregnancy), birth outcomes (at delivery) and perinatal mental health (antenatal and postpartum)”.

### Outcomes

Data from both CPRD and HES were used to ascertain the outcomes of interest. Pregnancy complications were identified using ICD-10 codes from HES records, as well as Read codes and SNOMED CT from CPRD clinical files. Additionally, the prescription of relevant medications was used to capture outcomes such as gestational hypertension, PE/eclampsia, GDM, and perinatal anxiety and depression. Pregnancy episodes in women with pre-existing diagnoses of hypertension, type 1 or 2 diabetes, anxiety, or depression recorded in CPRD at the start of pregnancy were excluded from the analysis of corresponding gestational outcomes (e.g., gestational hypertension, PE/eclampsia, GDM, perinatal anxiety, and perinatal depression). Other key outcomes included preterm birth, SGA, caesarean section, and stillbirth. Selection of Read, ICD-10 code lists was performed using an inhouse software platform called Code Builder [32], with systematic searching of existing code lists, and through clinical knowledge and discussion methods used in our previous publications. The definitions and list of codes used for outcome ascertainment are provided in additional file 2: Tables 1-14 and 15.

### Covariates

Covariates considered for adjustment include maternal age at the start of pregnancy (categorised into 5-year age bands), year of pregnancy, ethnicity, Index of Multiple Deprivation (IMD) in quintiles, latest BMI (Body mass index) prior to conception (or up to 16 weeks from the booking date if available), preconception smoking status (categorised as non-smoker, ex-smoker and current smoker), gravidity and a list of key comorbidities (hypertension, type 2 diabetes mellitus, anxiety, depression, psychosis). Ethnicity was identified using relevant Read codes from primary care records and was categorised as (1) White, (2) Asian, (3) Black and (4) mixed or multiple ethnic groups or (5) other ethnic groups. Primary care linked English IMD data provided a relative measure of deprivation based on seven different domains [36]. Pre-gravid BMI was identified as the latest BMI measured in primary care at least a year before the pregnancy start date and was categorised according to WHO (World health organisation) standards as underweight (< 18.5 kg/m^2^), normal weight (< 25 kg/m^2^), overweight (25–30 kg/m^2^) and obese (> 30 kg/m^2^). A separate missing category was created for those with missing data on ethnicity, deprivation, and pre-gravid BMI. The comorbidities were identified from primary care through relevant Read codes or Snomed CT codes.

### Statistical analysis

Baseline characteristics were described using median and interquartile range were for non-normally distributed continuous variables, and frequency and percentage for categorical variables, stratified by exposure of any autoimmune disease. Modified Poisson regression models with the log link function and robust standard errors to account for repeat pregnancies (from same women) was used to estimate relative risks (RR) and 95% confidence intervals (95% CI) for the association between autoimmune disease and outcomes of interest. Models were fitted for each autoimmune disease, for each outcome. Models were adjusted for potential confounders, demographic variables (age, ethnicity, socioeconomic status), clinical factors (smoking status, BMI, comorbidities), and gravidity. Missing data for ethnicity, deprivation, and pre-pregnancy BMI were handled using a missing-indicator approach; multiple imputation was not undertaken. Given the large number of autoimmune disease outcome comparisons and the potential correlation between tests, correction for multiple testing was performed using the Benjamini–Yekutieli (BY) procedure, which controls the false discovery rate under arbitrary dependence. The BY correction was applied separately within each pregnancy outcome, adjusting for the number of autoimmune diseases assessed per outcome. Associations were considered statistically significant if the BY-adjusted p-value was < 0.05. Analysis was performed in Stata IC version 17 (StataCorp) [9] and R studio (12.1) [37].

## Results

Total of 5,239,383 pregnancies in the CPRD pregnancy register and 2,485,366 births in the HES maternity records met the data quality criteria. Among them 188,682 pregnancies of 106,024 women in pregnancy cohort and 84,221 births of 63,972 women had a diagnosis of autoimmune diseases. The cohort selection is enumerated in flowchart 1 and 2. Baseline characteristic tabulated in Table 1.

Women with autoimmune disease were older at the time of pregnancy compared to women without autoimmune disease; median 31.1 vs. 29.7 years. Women with autoimmune diseases were more likely to be affluent (IMD 5 (least deprived quintile): 15.8% vs. 14.5%), white (57.1% vs. 49.8%)) have a higher BMI and gravidity. All the comorbidities studied were higher in the cohort of pregnancies of women with autoimmune diseases.

**Table 1:** Baseline characteristics of women aged 15–49 years in the UK (pregnancy cohort and birth cohort from CPRD Gold and CPRD Aurum)

Characteristics	Pregnancy cohort		Birth cohort	
	Autoimmune conditions (any)	No autoimmune conditions	Autoimmune conditions (any)	No autoimmune conditions
Number of pregnancies n (%)	188,602 (3.60)	5,050,781 (96.40)	84,221 (3.39)	2,401,145 (96.61)
Number of women n (%)	106,024 (3.70)	2,757,839 (96.30)	63,972 (3.50)	1,766,322 (96.50)
Maternal Age, median (IQR) years	31.11 (26.88–35.11)	29.70 (25.29–29.70)	30.68 (26.9-34.71)	29.73 (25.7-33.93)
Age categories n (%)				
>=15- <20 years	8,243 (4.36)	328,023 (6.49)	2,086 (2.48)	85,439 (3.56)
>=20- <25 years	22,985 (12.19)	812,828 (16.09)	8,061 (9.57)	298,453 (12.43)
>=25- <30 years	41,474 (21.99)	1,215,461 (24.06)	16,621 (19.73)	514,120 (21.41)
>=30- <35 years	56,965 (30.20)	1,429,576 (28.25)	23,859 (28.33)	627,939 (26.15)
>=35- <40 years	41,222 (21.86)	916,457 (18.14)	15,804 (18.76)	366,763 (15.27)
>=40- <45 years	14,323 (7.59)	292,880 (5.80)	3,413 (4.05)	79,270 (3.30)
>=45–49 years	3,390 (1.80)	55,556 (1.10)	14,375 (17.07)	428,825 (17.86)
**Index of multiple deprivation**,** n (%)**				
1-Most deprived	33,851 (17.95)	985,446 (19.51)	17,610 (20.91)	549,792 (22.90)
2	31,982 (16.96)	904,674 (17.91)	16,955 (20.13)	505,506 (21.05)
3	29,680 (15.74)	781,574 (15.47)	16,120 (19.14)	447,408 (18.63)
4	30,270 (16.05)	747,569 (14.80)	16,280 (19.33)	436,762 (18.19)
5-Least deprived	29,825 (15.81)	732,911 (14.51)	16,684 (19.181	441,146 (18.37)
Missing	32,994 (17.49)	898,607 (17.79)	100 (0.12)	3,251 (0.14)
**Smoking status**				
Non-Smoker	90,434 (47.95)	2,567,029 (50.82)	41,150 (48.86)	1,221,622 (50.88)
Ex-Smoker	31,371 (16.63)	685,995 (13.58)	14,224 (16.98)	326,868 (13.61)
Current smoker	53,584 (28.41)	1,301,219 (25.76)	22,719 (26.84)	589,261 (24.54)
Missing	13,213 (7.01)	496,538 (9.83)	6,128 (7.28)	263,394 (10.97)
**Body mass index (kg/m**^**2**^**)**,** n (%)**				
Median (IQR)	24.30 (21.60-28.39)	24.00 (21.36–28.10)	21.9 (4.76)	21.79 (4.73)
Underweight (< 18.5)	6,204 (3.29)	181,028 (3.58)	2,824 (3.35)	87,447 (3.64)
Normal weight (18.5-<25)	80,989 (42.94)	2,085,036 (41.28)	37,461 (44.48)	1,022,709 (42.59)
Overweight (25-<30)	39,794 (21.10)	977,645 (19.36)	17,617 (20.92)	458,452 (19.09)
Obese (≥ 30)	29,918 (15.86)	713,881 (14.13)	12,370 (14.69)	313,019 (13.04)
Missing	31,697 (16.81)	1,093,191 (21.64)	14,029 (17.12)	519,518 (22.39)
**Ethnicity n (%)**				
White	107,842 (57.18)	2,491,247 (49.32)	72,265 (85.88)	1,895,715 (78.95)
Mixed	36,111 (19.15)	1,054,579 (20.88)	1,095 (1.30)	34,972 (1.46)
Others*	1,592 (0.84)	50,163 (0.99)	2,645 (3.14)	108,989 (4.54)
Black	4,100 (2.17)	211,540 (4.19)	1,840 (2.18)	121,533 (5.06)
Asian^	10,392 (5.51)	332,553 (6.58)	4,481 (5.37)	157,763 (6.57)
Missing	28,565 (15.15)	910,699 (18.03)	1,895 (2.25)	82,173 (3.42)
**Gravidity**^**#**^ **n (%)**				
1	38,963 (20.66)	1,189,134 (23.54)	34,146 (40.54)	1,024,635 (42.67)
2	43,139 (22.87)	1,198,035 (23.72)	28,001 (33.26)	771,875 (32.15)
3	36,029 (19.10)	952,115 (18.85)	9,137 (10.85)	251,911 (10.49)
4	25,825 (13.69)	654,587 (12.96)	2,835 (3.37)	76,748 (3.20)
5+	44,646 (23.67)	1,056,910 (20.93)	6,173 (7.73)	163,695 (6.82)
**Comorbidities n (%)**				
Hypertension	4,219 (2.24)	73,319 (1.45)	1,546 (2.24)	31,999 (1.60)
Type 2 diabetes mellitus	2,433 (1.29)	26,191 (0.52)	720 (0.85)	6,751 (0.28)
Mental health conditions				
Depression	33,833 (17.94)	659,928 (13.07)	3,911 (25.61)	82,748 (20.04)
Anxiety	24,010 (12.73)	468,689 (9.28)	46 (0.30)	818 (0.20)
Psychosis	1,024 (0.54)	20,526 (0.41)	32 (0.21)	955 (0.23)

Numbers of pregnancies form the unit of analysis. ^The Asian category consisted of participants with origin from all over Asia, including India, Pakistan, China, Cambodia, Thailand, Vietnam, Malaysia, Sri Lanka, Nepal, Bangladesh, Japan, or Taiwan. *The ‘other’ ethnicity category consisted of patients with native American, Middle Eastern or Polynesian origin. # Total number of pregnancies

### Exposure to any of the autoimmune conditions and risk of pregnancy outcomes

Women with at least one autoimmune disease had a statistically significant higher risk of all antenatal pregnancy complications studied. Autoimmune disease was associated with the following outcomes: PE/eclampsia aRR (adjusted relative risk) 1.30 (95% confidence intervals 1.26–1.34), gestational hypertension aRR 1.22 (1.18–1.26), GDM aRR 1.09 (1.05–1.14), hyperemesis gravidarum aRR 1.08 (1.05–1.11), miscarriage aRR 1.06 (1.05–1.08) and ectopic pregnancy aRR 1.04 (1.00-1.09). For obstetric outcomes, women with autoimmune diseases were more likely to have caesarean birth aRR 1.20 (1.18–1.21), preterm birth aRR 1.45 (1.41–1.48), stillbirth aRR 1.11 (1.00-1.23) and SGA aRR 1.15 (1.08–1.22).

Women with autoimmune disease had a higher risk of adverse mental health conditions during pregnancy; anxiety aRR 1.25 (1.21–1.31) and depression aRR 1.20 (1.19–1.22). Individual autoimmune diseases are explored as risk factors for pregnancy complications, and the results are shown in the forest plot stratified by outcomes of interest in Fig. 3 and additional file 2 Figs. 5-22 and Table 17.

**Fig. 3:**
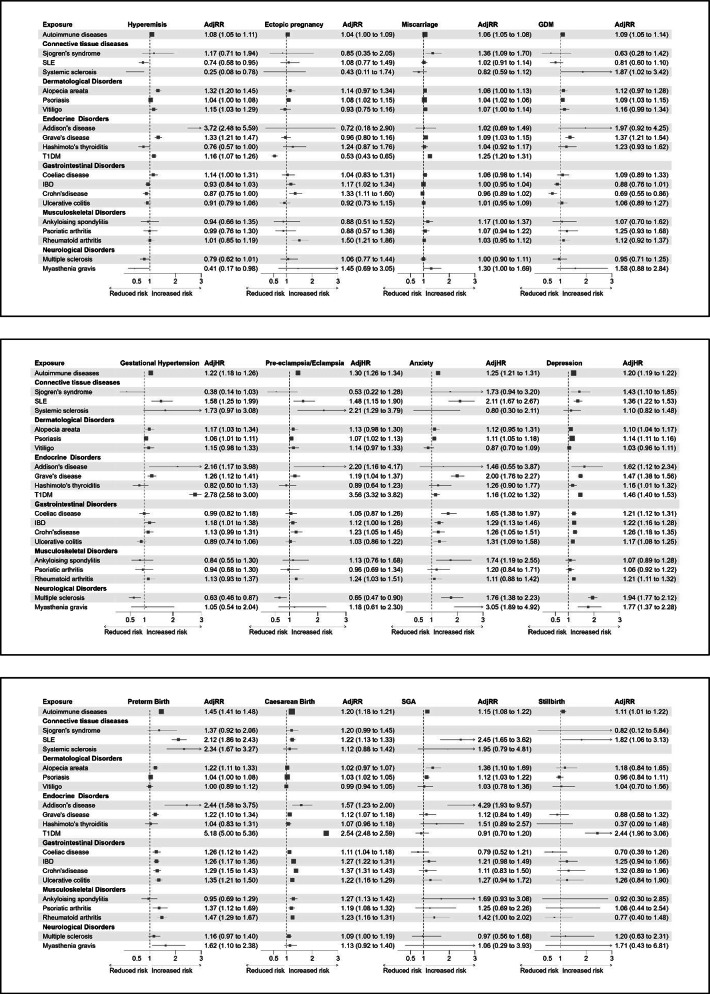
Forestplots reporting association of the autoimmune diseases and the adverse pregnancy outcomes. GDM= Gestational diabetes mellitus, SGA =small for gestational age, AdjRR=adjusted risk ratio. Analysis adjusted for maternal age, pregnancy year, ethnicity, IMD quintile, BMI, smoking status, gravidity, and comorbidities

### Systemic inflammatory disorders

Sjogren’s syndrome was associated with a higher risk of miscarriage aRR 1.66 (1.02–2.70) and depression during pregnancy aRR 1.43 (1.10–1.85). SLE was associated with an increased risk of gestational hypertension aRR 1.58 (1.25–1.99) and PE/eclampsia aRR 1.48 (1.15–1.90). Women with SLE also had an increased risk of total caesarean birth aRR 1.22 (1.13–1.33), including emergency aRR 1.24 (1.09–1.39) and elective aRR [1.20 (1.04–1.38)], nearly twice the risk for preterm birth aRR 2.12 (1.86–2.43), SGA aRR 2.45 (1.65–3.62) and stillbirth aRR 1.82 (1.12–1.84). Risk of anxiety and depression during pregnancy was also higher in women with SLE aRR 2.11 (1.67–2.67) and aRR 1.36 (1.22–1.53)], respectively. However, women with SLE had a 25% lower risk of hyperemesis gravidarum aRR 0.74 (0.58–0.95).

Women with systemic sclerosis had approximately two times greater risk of preterm birth aRR 2.34 (1.67–3.27) and GDM aRR 1.87 (1.02–3.42). Women with systemic sclerosis were at a 75% reduced risk of hyperemesis gravidarum aRR 0.25 (0.08–0.78) compared to women without systemic sclerosis.

### Dermatological diseases

Women with alopecia areata had a higher risk of hyperemesis gravidarum aRR 1.32 (1.20–1.45), miscarriage, aRR 1.06 (1.00-1.13) gestational hypertension aRR 1.17 (1.03–1.34), depression in pregnancy aRR 1.12 (1.00-1.27), aRR SGA 1.36 (1.22–1.53) and preterm birth aRR 1.25 (1.14–1.38).

Psoriasis in pregnancy was associated with a slightly higher risk of developing most of the outcomes studied; hyperemesis gravidarum aRR 1.04 (1.00-1.08), ectopic pregnancy aRR 1.08 (1.02–1.15), miscarriage aRR 1.04 (1.02–1.06), gestational hypertension, aRR 1.06 (1.01–1.11), PE/eclampsia aRR 1.07 (1.02–1.13), GDM aRR 1.09 (1.03–1.15), and SGA babies aRR 1.12 (1.03–1.22). Women with psoriasis were more likely to have a caesarean birth 1.02 (1.00-1.05) and preterm birth aRR 1.04 (1.00-1.08). Women with psoriasis also had a higher risk of perinatal mental health conditions; anxiety aRR 1.11 (1.05–1.18) and depression aRR 1.14 (1.11–1.16).

Women with vitiligo were more likely to develop hyperemesis gravidarum aRR 1.15 (1.03–1.29), or miscarriage aRR 1.07 (1.00-1.14).

### Endocrine disorders

Women with Addison’s disease had a four-fold higher risk of having SGA baby aRR 4.29 (1.93–9.57) and had two to three times higher risk of hyperemesis gravidarum aRR 3.72 (2.48–5.59), gestational hypertension aRR 2.16 (1.17–3.98), PE/eclampsia aRR 2.20 (1.16–4.17) and preterm birth aRR 2.44 (1.58–3.75). They were also at higher risk of caesarean birth aRR 1.50 (1.16–1.94).

Women with Graves’ disease had an increased risk of hyperemesis gravidarum aRR 1.33 [1.21–1.47), miscarriage aRR 1.09 (1.03–1.15), gestational hypertension aRR 1.26 (1.12–1.41), PE/eclampsia 1.19 (1.04–1.37), GDM aRR 1.37 (1.21–1.54), caesarean birth aRR 1.12 (1.07–1.18), and preterm birth aRR 1.23 (1.11–1.36). Graves’ disease was also associated with increased anxiety aRR 2.04 [1.80–2.32) and depression aRR 1.64 (1.46–1.83) during pregnancy. Hashimoto’s thyroiditis was linked to a higher risk of mental health conditions during pregnancy depression aRR 1.16 [1.01–1.32).

There was more than fivefold higher risk of preterm birth in mothers with T1DM, aRR 5.18 (5.00-5.36). There was also higher risk of caesarean birth aRR 2.50 (2.45–2.56) including both elective aRR 2.62 (2.52–2.74) and emergency aRR 2.39 (2.30–2.49). Pregnancies of women with T1DM were also around three times more likely to develop gestational hypertension, PE/eclampsia, and stillbirth aRR 2.78 (2.58-3.00), aRR 3.56 (3.32–3.82) and aRR 2.64 (2.09–3.33), respectively. Risk of miscarriage aRR 1.25 (1.20–1.31) hyperemesis gravidarum aRR 1.16 (1.07–1.26), anxiety aRR 1.16 (1.02–1.32) and depression aRR 1.46 (1.40–1.53) in pregnancy were also higher in women with T1DM. Risk of ectopic pregnancy was almost 50% lower in pregnancies of women with T1DM aRR 0.53 (043-0.65).

### Gastrointestinal diseases

Women with coeliac disease had a higher risk of developing hyperemesis gravidarum, anxiety and depression in pregnancy as compared to women without coeliac disease with aRR 1.14 (1.00-1.31), aRR 1.65 (1.38–1.97), and aRR 1.21 (1.12–1.31), respectively. The risk of caesarean birth aRR 1.11 (1.04–1.18) was higher in pregnancies of women with coeliac disease, and this was significantly higher for elective caesarean section aRR 1.16 (1.05–1.28) but not for emergency caesarean birth aRR 1.08 (0.99–1.17). There was also a 20% higher risk of preterm birth for women with coeliac disease compared to women without coeliac disease aRR 1.26 (1.12–1.42).

Women with IBD had an increased risk of ectopic pregnancy, with aRR of 1.17 (1.02–1.34), and of gestational hypertension aRR 1.18 (1.01–1.38), PE/eclampsia, aRR 1.12 (1.00-1.26). These risks were notably higher in women with Crohn’s disease; ectopic pregnancy aRR 1.33 (1.11–1.60), and PE/eclampsia aRR 1.23 (1.05–1.45). However, for women with ulcerative colitis, the associations were not statistically significant; ectopic pregnancy aRR 0.92 (0.73–1.15) and PE/eclampsia aRR 1.03 (0.86–1.22). Pregnancies in women with IBD also had a higher likelihood of caesarean birth, particularly elective, and were more likely to result in preterm birth, with aRR of 1.27 (1.22–1.31) and aRR 1.26 (1.17–1.36), respectively. The risk of anxiety or depression was elevated in women with IBD, with aRR 1.29 (1.13–1.46) and aRR 1.22 (1.16–1.28) respectively, and was similarly increased in both Crohn’s disease and ulcerative colitis. Interestingly, women with Crohn’s disease had 30% lower risk of GDM aRR 0.69 (0.55–0.86), although no significant associations with GDM were observed in women with ulcerative colitis or IBD overall compared to those without these conditions.

### Musculoskeletal disorders

There was almost a two-fold higher risk of developing anxiety in pregnancy aRR 1.78 (1.39–2.27) in women with ankylosing spondylitis. Risk of ectopic pregnancy and caesarean birth were also slightly higher in women with ankylosing spondylitis aRR 1.17 (1.00-1.37) and aRR 1.27 [1.13–1.42), respectively.

Women with psoriatic arthritis had a 50% greater risk of developing GDM aRR 1.44 (1.07–1.93). Higher risk for preterm birth aRR 1.37 (1.12–1.69) and caesarean birth aRR 1.19 (1.08–1.32), with a higher likelihood of emergency caesarean aRR 1.27 [1.11–1.46) was also observed.

Women with rheumatoid arthritis had a higher risk of hyperemesis gravidarum aRR 1.50 (1.21–1.86) and preterm birth aRR 1.53 [1.33–1.76). They were also more likely to develop PE/eclampsia aRR 1.24 [1.03–1.51), have a caesarean birth aRR 1.23 (1.16–1.31), have SGA babies aRR 1.42 (1.00-2.02) and develop depression during pregnancy aRR 1.21 (1.11–1.32).

### Neurological disorders

Women with multiple sclerosis had nearly double the risk of mental health conditions during pregnancy, depression aRR 1.94 (1.77–2.12) and anxiety aRR 1.76 (1.38–2.23). They also have higher risk of elective caesarean birth aRR 1.21 (1.06–1.39) and preterm birth aRR 1.25 (1.06–1.48). On the other hand, multiple sclerosis was inversely associated with gestational hypertension aRR 0.63 (0.54–0.87) and PE/eclampsia aRR 0.65 (0.47–0.90).

Women with myasthenia gravis had a markedly higher risk of anxiety aRR 3.05 (1.89–4.92) and depression aRR 1.77 (1.37–2.28) during pregnancy. They also experienced a 30% to 60% increased risk of miscarriage aRR 1.30 (1.00–1.69) and a 60% higher risk of preterm birth aRR 1.60, (1.04–2.46). In contrast, pregnancies in women with myasthenia gravis were less likely to be affected by hyperemesis gravidarum aRR 0.41 (0.17–0.98).

### Benjamini–Yekutieli correction

After Benjamini–Yekutieli correction for multiple testing, the number of statistically significant associations was reduced, with remaining signals representing a core set of robust findings. Significant associations persisted primarily for any autoimmune disease and a limited number of specific conditions, most consistently T1DM, SLE, Graves’ disease, and IBD across outcomes including hyperemesis gravidarum, ectopic pregnancy, hypertensive disorders of pregnancy, gestational diabetes, perinatal mental health outcomes, preterm birth, caesarean birth, stillbirth, and small for gestational age. Associations for several other autoimmune conditions that were nominally significant did not remain so after correction and should be interpreted as hypothesis-generating. Full results of the multiple-testing–corrected analyses are presented in Supplementary Table 18.

## Discussion

### Principal findings

Our study reports significant associations between autoimmune diseases and a wide range of adverse pregnancy outcomes, which were maintained after applying the Benjamini–Yekutieli correction for multiple testing. For early pregnancy outcomes, hyperemesis gravidarum was associated with alopecia areata, Addison’s disease, Graves’ disease, and T1DM.Ectopic pregnancy was associated with rheumatoid arthritis and Crohn’s disease. Miscarriage was associated with psoriasis, IBD, and ulcerative colitis. For hypertensive disorders, gestational hypertension was associated with SLE, psoriasis, Graves’ disease, T1DM, and autoimmune diseases overall, whereas no statistically significant associations were observed for pre-eclampsia or eclampsia after correction. Gestational diabetes mellitus was associated with psoriasis, Graves’ disease, and Crohn’s disease. The most consistent associations were observed for perinatal mental health outcomes, with several autoimmune diseases including SLE, psoriasis, Graves’ disease, IBD, Crohn’s disease, ulcerative colitis, multiple sclerosis, and myasthenia gravis associated with increased risks of perinatal anxiety and/or depression.

For birth outcomes, preterm birth and caesarean delivery were associated with a broad range of autoimmune diseases, while associations with small for gestational age were observed primarily for SLE, T1DM, and multiple sclerosis. No statistically robust associations were identified for stillbirth following correction, likely reflecting limited power for this rare outcome.

This study makes three key contributions to the existing literature. First, it provides comprehensive, condition-specific estimates across a wide range of autoimmune diseases and pregnancy outcomes within a single, harmonised population-based framework, allowing direct comparison of risks across conditions and outcome domains. Second, by applying correction for multiple testing, it identifies a core set of associations, addressing a key limitation of prior studies. Third, it extends the evidence base to less commonly studied autoimmune diseases, including Addison’s disease and myasthenia gravis, and highlights perinatal mental health outcomes as an affected outcome across multiple autoimmune conditions.

### Results in the context of what is known

Our findings are broadly consistent with existing evidence demonstrating increased risks of adverse pregnancy outcomes among women with autoimmune diseases. However, unlike previous studies that have largely focused on single conditions or limited outcome sets, this study provides a systematic comparison across multiple autoimmune diseases and outcome domains within the same population. This study extends the literature by reporting associations for less commonly studied autoimmune conditions, including Addison’s disease, myasthenia gravis, and ankylosing spondylitis. Sjögren’s syndrome, which predominantly affects women [38], has been relatively understudied in pregnancy but has been associated with miscarriage [39]. Established associations between SLE and miscarriage and hypertensive disorders of pregnancy [10, 11, 40] were confirmed.

Consistent with previous studies, psoriasis was associated with miscarriage and gestational hypertension or pre-eclampsia, although no association with GDM was observed [41, 42]. Less commonly studied dermatological conditions, including alopecia areata and vitiligo, were also associated with miscarriage and gestational hypertension, supporting earlier observational findings [43–45]. A reduced risk of hyperemesis gravidarum observed in women with systemic sclerosis, SLE, and myasthenia gravis represents a novel finding that warrants further investigation.

Among endocrine autoimmune diseases, T1DM has been extensively studied and was associated with gestational hypertension, in line with previous evidence [46]. An inverse association with ectopic pregnancy was also observed, which requires further exploration Graves’ disease and Hashimoto’s thyroiditis have been less well studied in pregnancy; existing literature suggests thyroid autoimmunity is associated with miscarriage and gestational hypertension [47], and this study provides condition-specific estimates. Evidence on pregnancy outcomes in Addison’s disease is limited, although increased obstetric risk has been reported [48], our findings extend this evidence to include hyperemesis gravidarum, gestational hypertension, GDM, and depression.

Rheumatoid arthritis is the most extensively studied musculoskeletal autoimmune disease, and previously reported associations with miscarriage, gestational hypertension, and GDM were confirmed [49, 50]. While earlier studies reported no clear associations for ankylosing spondylitis [51], this study identified increased risks of miscarriage and anxiety in pregnancy. Previous reviews suggested increased hypertension risk but inconclusive evidence for GDM in psoriatic arthritis [42, 52] in contrast, we observed an increased risk of GDM without an association with hypertension. For gastrointestinal autoimmune diseases, previous associations between coeliac disease and miscarriage [53] and between IBD and GDM have been reported [54], our findings support the miscarriage association but not GDM and demonstrate increased risks of perinatal mental health conditions. Evidence for multiple sclerosis has been limited and inconsistent [55, 56], this study demonstrates increased perinatal mental health risk and a novel inverse association with hypertensive disorders. Limited literature on myasthenia gravis suggests an association with pre-eclampsia/eclampsia [57], which was not observed; however, increased risks of anxiety and depression were identified. Elevated perinatal mental health risks were also observed in women with coeliac disease, IBD, and T1DM.

### Research implications

The mechanisms behind adverse pregnancy outcomes are complex, involving physiological changes, treatment regimens, and immune responses. Chronic inflammation and immune dysregulation can impair placental function, increasing the risks of PE/eclampsia, fetal growth restriction, premature birth, and low birth weight [58].

Conditions such as IBD, coeliac disease, and multiple sclerosis further disrupt placental function through cytokine imbalances and inflammation, with nutritional deficiencies exacerbating these risks [59]. Increased proinflammatory cytokines in autoimmune conditions like psoriasis and vitiligo disrupt immune balance during pregnancy, leading to higher rates of miscarriage and lower live birth rates [60, 61].

Hyperglycaemia in T1DM induces oxidative stress and immune dysregulation, heightening the likelihood of PE/eclampsia and miscarriage [62, 63]. Thyroid autoimmunity negatively impacts the fetal-maternal interface, resulting in miscarriage and premature birth due to altered cytokine expression and thyroid hormone levels [64]. Additionally, Addison’s disease is associated with preterm birth due to insufficient cortisol replacement therapy [65].

The treatment of autoimmune conditions during pregnancy requires balancing inflammation control with potential risks to both mother and fetus. Corticosteroids, while effective at managing inflammation, can increase the risk of GDM, hypertension, pre-eclampsia, and intrauterine growth restriction (IUGR) [66, 67]. Some immunosuppressive drugs such as methotrexate or mycophenolate mofetil are contraindicated in pregnancy and may increase the risk including preterm birth, low birth weight, congenital abnormalities, and neonatal complications such as immunosuppression or infections [68–71]. Therefore, careful consideration of treatment options is essential, with a thorough risk-benefit analysis weighing medication exposure against the consequences of untreated inflammation. Involving the healthcare team, the patient, and their support network in discussions ensures that both maternal and fetal risks are properly addressed [72]. While biological mechanisms such as immune dysregulation and inflammation likely underpin these associations, the key contribution of this study is to provide robust epidemiological evidence to prioritise which autoimmune conditions and outcomes warrant further mechanistic and interventional research.

### Clinical implications

Despite existing guidelines, there remains limited integration of condition-specific risk estimates across a broad range of pregnancy outcomes in women with autoimmune diseases [73–77], many focus primarily on disease control and medication use, with less emphasis on the broader range of potential pregnancy complications [78–83]. Our findings may help to inform future guideline development by highlighting associations between specific autoimmune conditions and adverse obstetric and perinatal outcomes, including hypertensive disorders and perinatal mental health conditions. In particular, the observed associations with anxiety and depression underscore the importance of considering psychosocial wellbeing alongside physical health in antenatal care for women with autoimmune diseases.

While this observational study cannot inform treatment strategies, it supports the need for heightened clinical awareness, multidisciplinary care, and tailored counselling for women with autoimmune conditions during pregnancy. Further research is warranted to elucidate underlying mechanisms and to determine whether targeted monitoring or interventions could reduce the risks identified.

### Strengths and limitations

Key strengths of this study include the use of a large, nationally representative cohort of 106,024 women with autoimmune diseases drawn from the CPRD pregnancy register, linked to Hospital Episode Statistics. This provided rich longitudinal data across primary and secondary care, enabling adjustment for a wide range of demographic, socioeconomic, and clinical confounders, including age, deprivation, and comorbidities. An additional major strength is that this study extends the existing literature by reporting associations for several less commonly studied autoimmune conditions, including Addison’s disease, myasthenia gravis, and ankylosing spondylitis. Sjögren’s syndrome, which predominantly affects women [84], has also been relatively understudied in pregnancy, and this study provides new population-based evidence on its associations with adverse pregnancy outcomes. Rigorous eligibility and quality-control procedures were applied to improve the validity of pregnancy ascertainment, including exclusion of overlapping or implausible pregnancy episodes, insufficient registration history, and records lacking an acceptable patient flag.

Several limitations should be acknowledged. As an observational analysis of routinely collected healthcare data, causal inference is limited and residual confounding from unmeasured or incompletely captured factors may persist. Autoimmune disease status was identified using routinely recorded diagnostic codes in CPRD-HES; coding accuracy may vary across conditions, particularly for rarer diseases, and changes in diagnostic criteria and recording practices over the 2000–2022 study period may have contributed to exposure misclassification. Although all models were adjusted for calendar year of pregnancy, the absence of formal time-stratified analyses means that pooled estimates may reflect average associations across periods with differing baseline risks and clinical practices.

Detailed information on disease activity, severity, serological status, organ involvement, flares, and immunomodulatory medication use, including drug type, dose, adherence, and timing relative to conception was unavailable. Consequently, estimates represent average associations across heterogeneous disease states, and confounding by indication may partly explain some findings. For several rare autoimmune diseases, such as myasthenia gravis and Addison’s disease, case numbers were small; while relative effect estimates were sometimes large, absolute risks are likely to be low and should be interpreted with caution. Early pregnancy loss and perinatal mental health outcomes may be under-ascertained, as very early miscarriages often do not present to healthcare services and mental health conditions during pregnancy are frequently under-recognised. In addition, use of a missing-indicator approach for covariates may not fully address systematic differences between individuals with complete and incomplete data, and some residual bias may remain. Finally, as the study was conducted within the UK NHS primary care setting with linkage to HES, findings may not be fully generalisable to populations with different healthcare systems, ethnic compositions, or underlying disease prevalence.

Future research should corroborate these findings using prospective study designs with detailed phenotyping, including disease activity, severity, and medication exposure, to better elucidate causal pathways and underlying biological mechanisms.

## Conclusions

This study demonstrates that autoimmune diseases are associated with a wide spectrum of adverse pregnancy outcomes, with considerable variation in risk across individual conditions. Through a systematic evaluation of multiple autoimmune diseases across key outcome domains, including early pregnancy, obstetric, metabolic, and perinatal mental health outcomes this analysis enables direct comparison of risk patterns within a single population-based framework. The findings reveal both shared risks across several conditions and distinct, condition-specific associations, with perinatal mental health outcomes emerging as a consistently elevated risk across diverse autoimmune diseases. By generating robust, population-level estimates for both common and less frequently studied conditions, this study strengthens the evidence base and provides a clearer foundation for risk stratification, clinical care, and future research.

## Supplementary Information

Below is the link to the electronic supplementary material.

Supplementary Material 1: Additional file 1

Supplementary Material 2: Additional file 2

## Data Availability

Access to anonymized patient data from CPRD is subject to a data sharing agreement containing detailed terms and conditions of use following protocol approval from the MHRA Independent Scientific Advisory Committee. This study-specific analysable dataset is therefore not publicly available but can be requested from the corresponding author at [f.crowe@bham.ac.uk](mailto: f.crowe@bham.ac.uk) subject to research data governance approvals. Details about Independent Scientific Advisory Committee applications and data costs are available on the CPRD website (cprd.com).

## Abbreviations

Abbreviation: Full Term
AID: Autoimmune disease
aRR: Adjusted relative risk
BY: Benjamini–Yekutieli (procedure)
CI: Confidence interval
CPRD: Clinical Practice Research Datalink
EMIS: Egton Medical Information Systems
GDM: Gestational diabetes mellitus
HES: Hospital Episode Statistics
IBD: Inflammatory bowel disease
ICD-10: International Classification of Diseases, 10th Revision
MS: Multiple sclerosis
PE: Pre-eclampsia
PTB: Preterm birth
RR: Relative risk
SGA: Small for gestational age
SLE: Systemic lupus erythematosus
SNOMED: Systematized Nomenclature of Medicine
T1DM: Type 1 diabetes mellitus
