# Supplementary material for: Autoimmune diseases and risk of adverse pregnancy outcomes: a population-based cohort study of five million pregnancies in the UK
Source: BMC Med. 2026 May 21;24:398. doi: 10.1186/s12916-026-04921-w (PMC13374351; doi:10.1186/s12916-026-04921-w)
Supplement: Supplementary file 2 — Supplementary Material 2: Additional file 2 [file 12916_2026_4921_MOESM2_ESM.docx]

Contents

[Contents 1](#_Toc227828416)

[Figure 1 Flowchart of the study selection for CPRD Gold pregnancy cohort 3](#_Toc227828417)

[Figure 2 Flowchart of the study selection for CPRD Aurum pregnancy cohort 4](#_Toc227828418)

[Figure 3 Flowchart of the study selection for CPRD Aurum maternity tail 5](#_Toc227828419)

[Figure 4 Flowchart of the study selection for CPRD GOLD maternity tail 6](#_Toc227828420)

[Table 1 Definition of Hyperemesis gravidarum 6](#_Toc227828421)

[Table 2 Definition of Ectopic pregnancy 6](#_Toc227828422)

[Table 3 Definition of Miscarriage 7](#_Toc227828423)

[Table 4Definition of Gestational hypertension 7](#_Toc227828424)

[Table 5 Definition of Pre-eclampsia/Eclampsia 7](#_Toc227828425)

[Table 6 Definition of Gestational diabetes 8](#_Toc227828426)

[Table 7Definition of antenatal anxiety 8](#_Toc227828427)

[Table 8Definition of antenatal depression 8](#_Toc227828428)

[Table 9 Definition of postnatal anxiety 9](#_Toc227828429)

[Table 10 Definition of postnatal depression 9](#_Toc227828430)

[Table 11 Definition of Preterm birth 9](#_Toc227828431)

[Table 12 Definition of Small for gestational age 10](#_Toc227828432)

[Table 13Definition of Caesarean section 10](#_Toc227828433)

[Table 14 Definition of Stillbirth 10](#_Toc227828434)

[Table 15 Clinical codes for the outcomes 10](#_Toc227828435)

[Figure 5 Forest plot describing association of autoimmune diseases in women and Hyperemesis gravidarum 27](#_Toc227828436)

[Figure 6 Forest plot describing association of autoimmune diseases in women and Ectopic pregnancy 28](#_Toc227828437)

[Figure 7 Forest plot describing association of autoimmune diseases in women and Miscarriage 29](#_Toc227828438)

[Figure 8 Forest plot describing association of autoimmune diseases in women and Gestational hypertension 30](#_Toc227828439)

[Figure 9 Forest plot describing association of autoimmune diseases in women and Pre-eclampsia 32](#_Toc227828440)

[Figure 10 Forest plot describing association of autoimmune diseases in women and Gestational diabetes mellitus 33](#_Toc227828441)

[Figure 11 Forest plot describing association of autoimmune diseases in women and Antenatal anxiety 34](#_Toc227828442)

[Figure 12 Forest plot describing association of autoimmune diseases in women and Antenatal depression 35](#_Toc227828443)

[Figure 13 Forest plot describing association of autoimmune diseases in women and postnatal anxiety 36](#_Toc227828444)

[Figure 14 Forest plot describing association of autoimmune diseases in women and postnatal depression 36](#_Toc227828445)

[Figure 15 Forest plot describing association of autoimmune diseases in women and Caesarean section 37](#_Toc227828446)

[Figure 16 Forest plot describing association of autoimmune diseases in women and Elective Caesarean section 38](#_Toc227828447)

[Figure 17 Forest plot describing association of autoimmune diseases in women and Emergency Caesarean section 39](#_Toc227828448)

[Figure 18 Forest plot describing association of autoimmune diseases in women and Preterm birth 40](#_Toc227828449)

[Figure 19 Forest plot describing association of autoimmune diseases in women and Small for gestational age 41](#_Toc227828450)

[Figure 20 Forest plot describing association of autoimmune diseases in women and Stillbirth 43](#_Toc227828451)

[Figure 21 Forest plot describing association of autoimmune diseases in women and Perinatal anxiety 43](#_Toc227828452)

[Figure 22 Forest plot describing association of autoimmune diseases in women and Perinatal depression 44](#_Toc227828453)

[Table 17 Heat Map-diseases and adverse pregnancy outcomes 44](#_Toc227828454)

[Table 18 Raw adjusted and Benjamini–Yekutieli–corrected p-values for associations between autoimmune diseases and pregnancy complications. 45](#_Toc227828455)


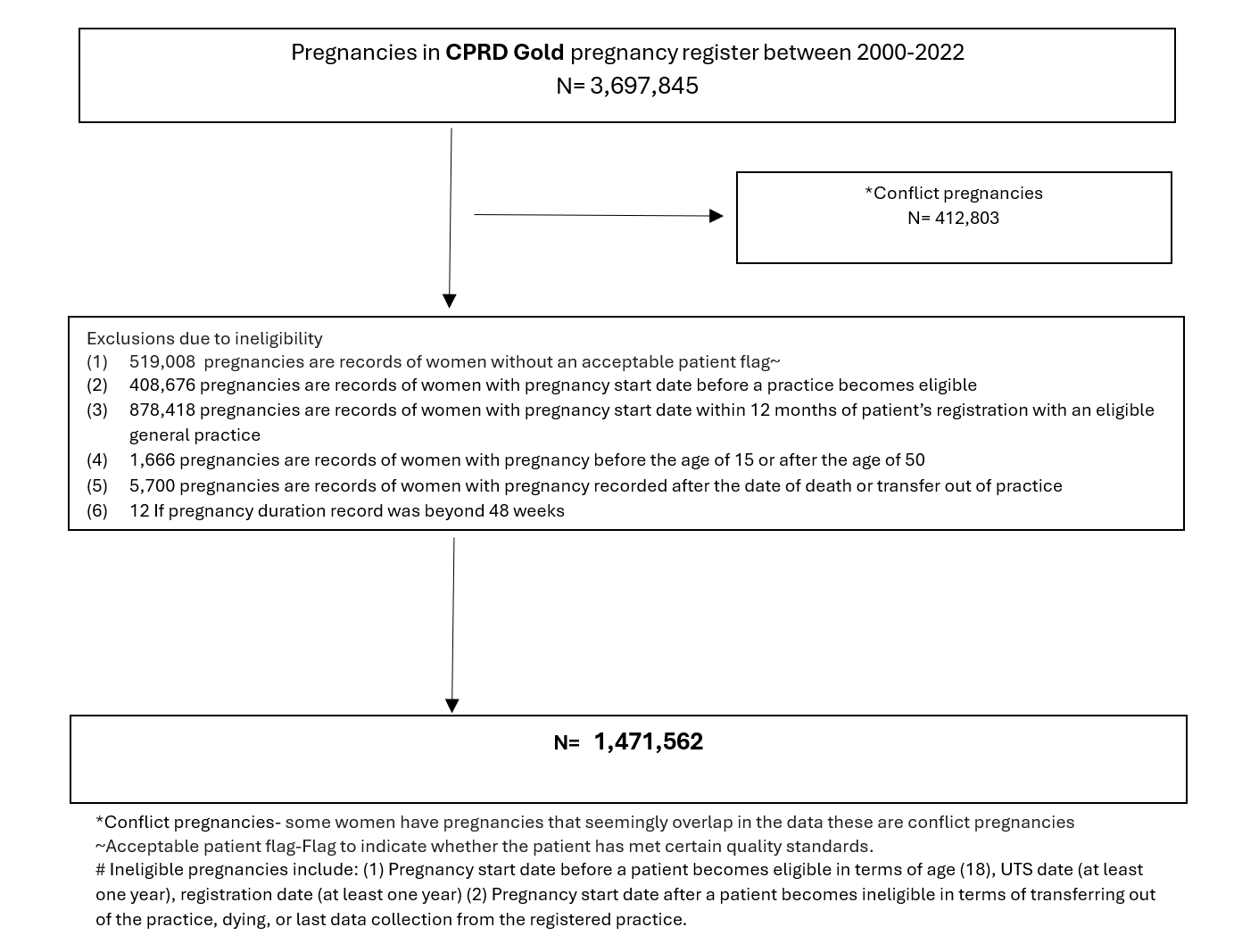


# Figure 1 Flowchart of the study selection for CPRD Gold pregnancy cohort


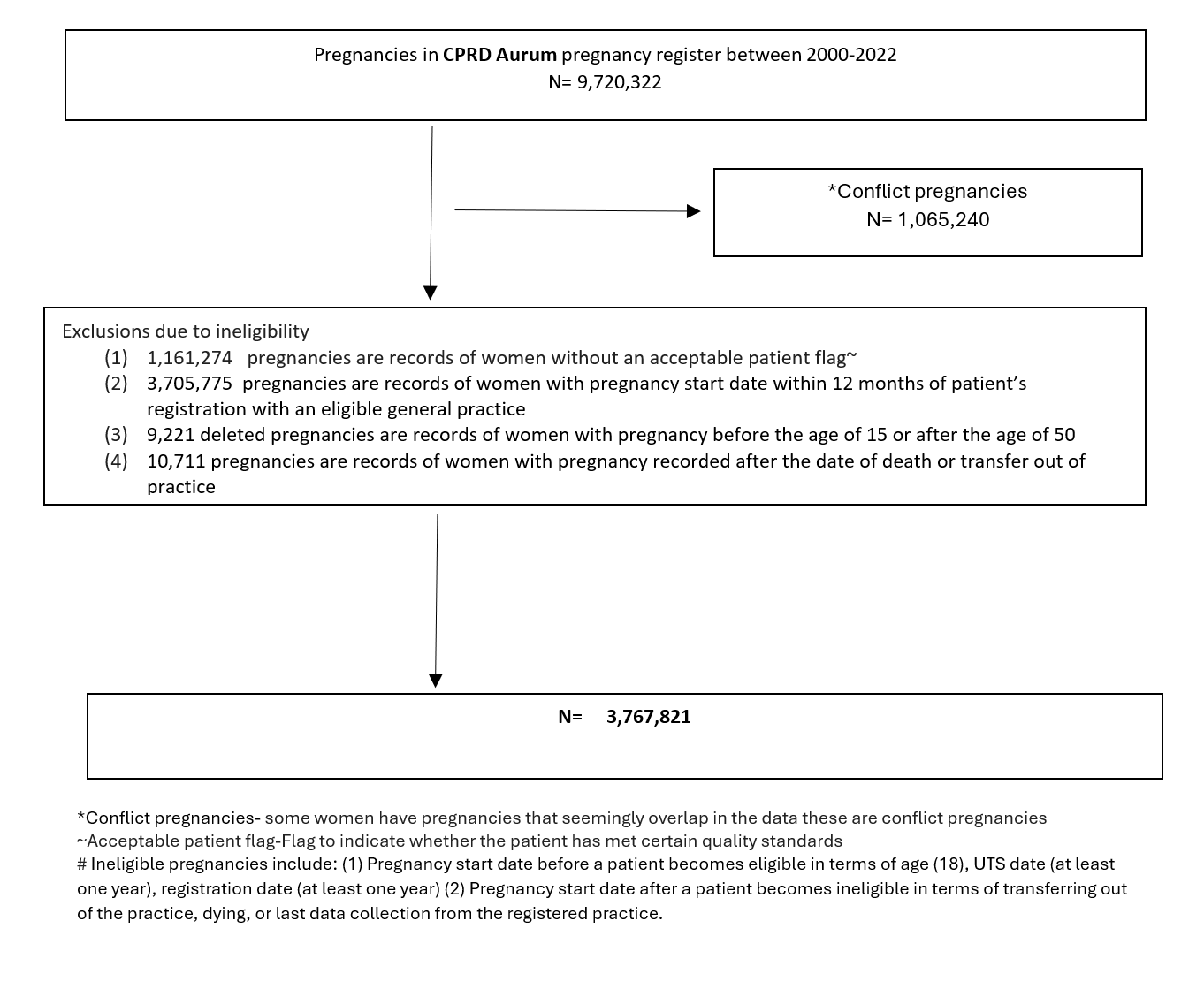


# Figure 2 Flowchart of the study selection for CPRD Aurum pregnancy cohort


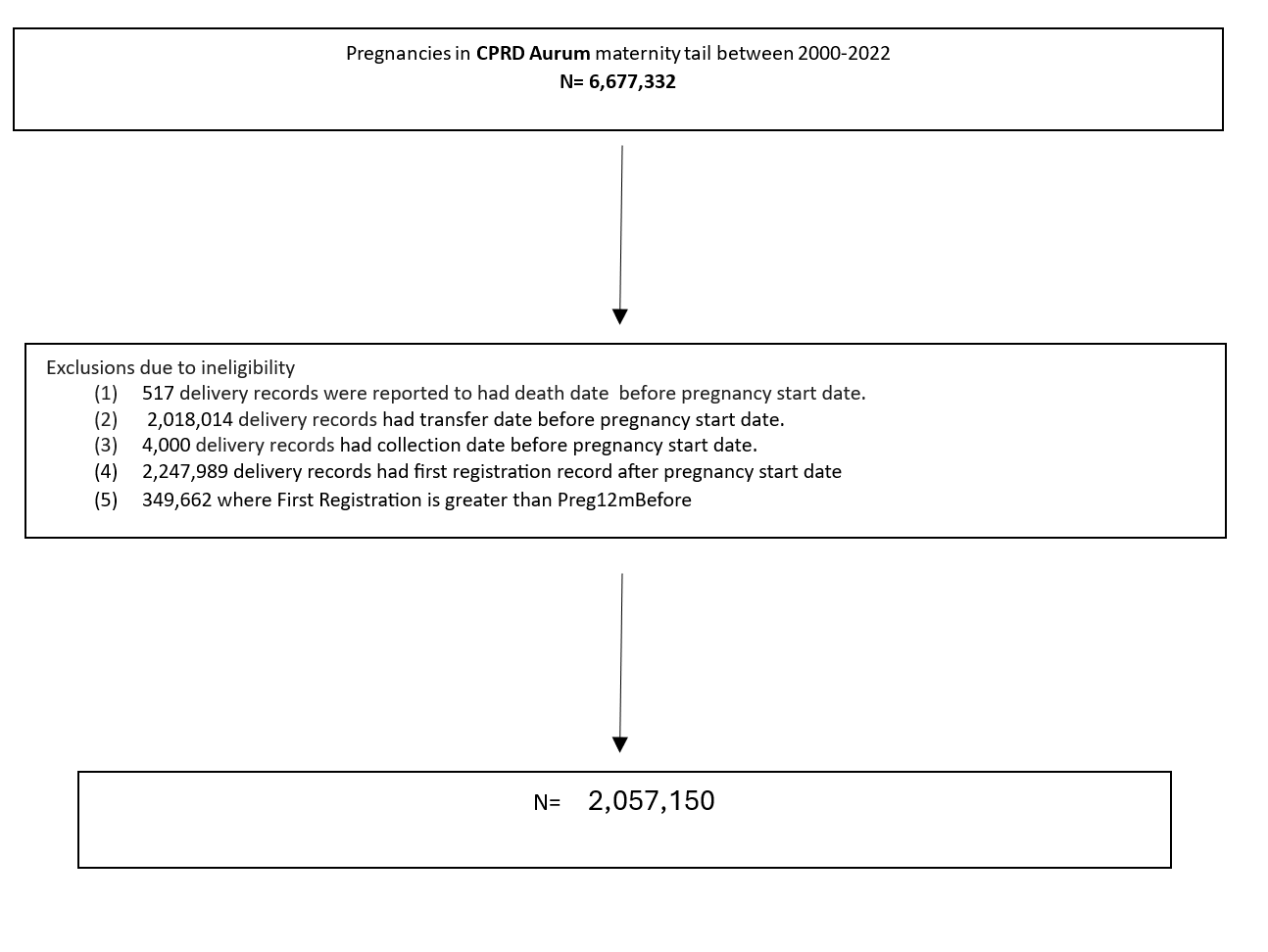


# Figure 3 Flowchart of the study selection for CPRD Aurum maternity tail


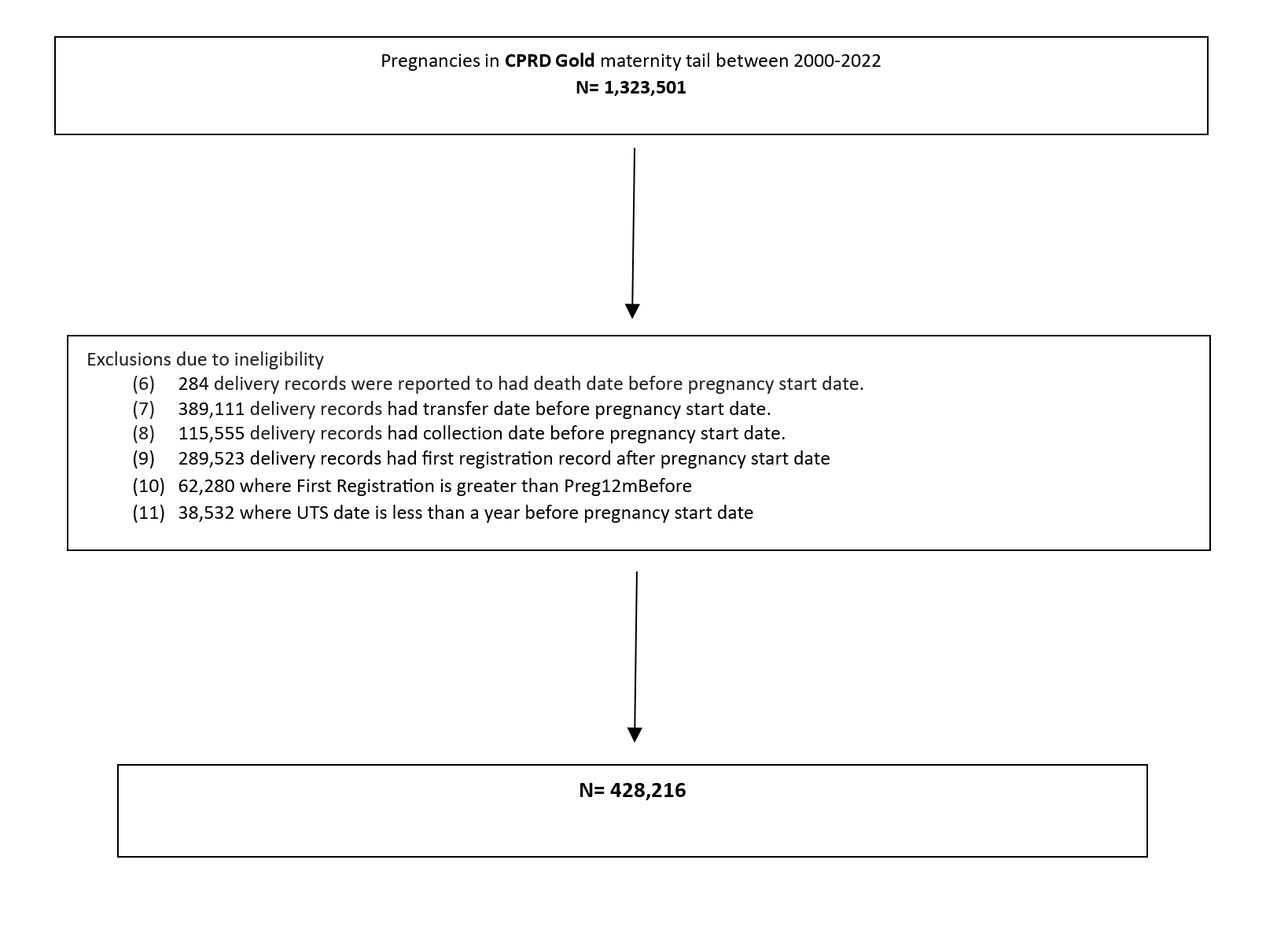


# Figure 4 Flowchart of the study selection for CPRD GOLD maternity tail

# Table 1 Definition of Hyperemesis gravidarum

| Time frame | Base cohort |
| --- | --- |
| Pregnancy start date- 42 weeks | Primary care Primary care-Read codes/SNOMED CT codes |
| Pregnancy start date- 42 weeks | Secondary care=ICD codes |

# Table 2 Definition of Ectopic pregnancy

| Time Frame | Base cohort |
| --- | --- |
| Pregnancy start date-30 weeks | Primary care Read codes/SNOMED CT codes |
| Pregnancy start date-30 weeks | Secondary care-ICD 10 codes |
| No timeline | Pregnancy register Flag==7 |

# Table 3 Definition of Miscarriage

| Time frame | Base cohort |
| --- | --- |
| Pregnancy start date-24 weeks +6 weeks | Primary care-Read codes/SNMOMED CT code |
| Pregnancy start date-24 weeks +6 weeks | Secondary care=ICD codes |
| No timeline | Pregnancy register Flag==4 |
|  |  |

# Table 4Definition of Gestational hypertension

| Exclude | Before Pregnancy start date+20*7  Read codes/SNOMED CT codes | Hypertension diagnosis in primary and secondary care Drug codes (Thiazides, caicium channel blockers, Ace inhibitors, ARB’s, Aliskiren alpha blocker) |
| --- | --- | --- |
| Base cohort  CPRD Gold N=1,455,922  CPRD Aurum N= 3,684,536 | | |
| Include | Pregnancy start date+20 weeks upto Pregnancy end date +6weeks | Diagnostic codes for hypertension, gestational hypertension, pre-eclampsia or eclampsia  Additionally drugs codes for (amlodipine, doxazosin, labetolol, methyldopa, nifedipine) |
| Include | Pregnancy start date+20 weeks upto Pregnancy end date +6weeks | Secondary care=ICD codes  Gestational hypertension, pre-eclampsia, eclampsia |

# Table 5 Definition of Pre-eclampsia/Eclampsia

| Exclude | Before Pregnancy start date+20*7  Read codes/SNOMED CT codes | Hypertension diagnosis in primary and secondary care Drug codes (Thiazides, caicium channel blockers, Ace inhibitors, ARB’s, Aliskiren alpha blocker) |
| --- | --- | --- |
| Base cohort  CPRD Gold N=1,455,922  CPRD Aurum N= 3,684,536 | | |
| Include | Pregnancy start date+20 weeks upto Pregnancy end date +6weeks | Diagnostic codes for pre-eclampsia or eclampsia  Additionally drugs codes for (amlodipine, doxazosin, labetolol, methyldopa, nifedipine) |
| Include | Pregnancy start date+20 weeks upto Pregnancy end date +6weeks | Secondary care=ICD codes  pre-eclampsia, eclampsia |

# Table 6 Definition of Gestational diabetes

| Exclude | Before Pregnancy start date+20*7  Read codes/SNOMED CT codes | T1DM&T2DM diagnosis in primary and secondary care Drug codes (Acarbose, Meglitinides, Insulin, GLP-1, DPP4inhibitors) |
| --- | --- | --- |
| Base cohort  CPRD Gold N=1,455,922  CPRD Aurum N= 3,684,536 | | |
| Include | Pregnancy start date+20 weeks upto Pregnancy end date +6weeks | Diagnostic codes for gestational diabetes  Additionally drugs codes for insulin, metformin |
| Include | Pregnancy start date+20 weeks upto Pregnancy end date +6weeks | Secondary care=ICD codes  Gestational diabetes |

# Table 7Definition of antenatal anxiety

| Exclude | Before Pregnancy start date  Read codes/SNOMED CT codes | Anxiety diagnosis in primary and secondary care Drug codes (Anxiolyticts, propranolol, IAPT codes) |
| --- | --- | --- |
| Base cohort  CPRD Gold N= 1,303,681  CPRD Aurum N= 3,275,122 | | |
| Include | Pregnancy start date upto Pregnancy end date +6weeks | Diagnostic codes for anxiety  Additionally drugs codes for anxiolytics |
| Include | Pregnancy start date upto Pregnancy end date +6weeks | Secondary care=ICD codes  anxiety |

# Table 8Definition of antenatal depression

| Exclude | Before Pregnancy start date  Read codes/SNOMED CT codes | Depression diagnosis in primary and secondary care Drug codes (propranolol, SSRI, TCAdepressants, MAOI, other antidepressants, IAPT codes) |
| --- | --- | --- |
| Base cohort  CPRD Gold N= 1,267,141  CPRD Aurum N= 3,074,060 | | |
| Include | Pregnancy start date upto Pregnancy end date +6weeks | Diagnostic codes for depression  Additionally drugs codes for (propranolol, SSRI, TCAdepressants, MAOI, other antidepressants, IAPT codes) |
| Include | Pregnancy start date upto Pregnancy end date +6weeks | Secondary care=ICD codes  depression |

# Table 9 Definition of postnatal anxiety

| Exclude | Before Pregnancy end date  Read codes/SNOMED CT codes | Anxiety diagnosis in primary and secondary care Drug codes (Anxiolyticts, propranolol, IAPT codes |
| --- | --- | --- |
| Base cohort  CPRD Gold N= 1,298,290  CPRD Aurum N= 3,259,742 | | |
| Include | Pregnancy end date + 1year | Diagnostic codes for anxiety  Additionally drugs codes for anxiolytics |
| Include | Pregnancy end date + 1year | Secondary care=ICD codes  anxiety |

# Table 10 Definition of postnatal depression

| Exclude | Before Pregnancy end date  Read codes/SNOMED CT codes | Depression diagnosis in primary and secondary care Drug codes (propranolol, SSRI, TCAdepressants, MAOI, other antidepressants, IAPT codes) |
| --- | --- | --- |
| Base cohort  CPRD Gold N= 1,259,932  CPRD Aurum N= 3,059,618 | | |
| Include | Pregnancy end date + 1year | Diagnostic codes for depression  Additionally drugs codes for (propranolol, SSRI, TCAdepressants, MAOI, other antidepressants, IAPT codes) |
| Include | Pregnancy end date + 1year | Secondary care=ICD codes  depression |

# Table 11 Definition of Preterm birth

| Time frame | Base cohort |
| --- | --- |
| Delivery cohort | Gestational age |
| HES linked | Secondary care=ICD codes |

# Table 12 Definition of Small for gestational age

| Time frame | Base cohort |
| --- | --- |
| Delivery cohort | Birthweight below the 10th percentile for gestational age and sex, based on international standards. Birthweight, gestational age, and sex were entered into INTERGROWTH-21st project software to calculate weight-for-gestational-age centiles. |

# Table 13Definition of Caesarean section

| Time frame | Base cohort |
| --- | --- |
| Delivery cohort | Calculated from Delivery method variable in maternity tail and OPCS coded in HES linked data |

# Table 14 Definition of Stillbirth

| Time frame | Base cohort |
| --- | --- |
| Delivery cohort | Stillbirth was defined using the birth status variable from the maternity tail |

# Table 15 Clinical codes for the outcomes

|  | CPRD Gold | CPRD Aurum | ICD 10 |
| --- | --- | --- | --- |
| Miscarriage | L04..11  L02..00  L040111  L045x00  L042x00  L043y00  L040100  L040w00  L041x00  L043x00  L043x11  L042000  L040400  L040x00  L045100  L043z11  L042100  L045211  L045111  L045x11  L043y11  L044111  L043100  L041200  L044000  L044200  L10zz11  L04..00  7E08800  L010.00  L044.00  L041.00  L042.00  L043.00  1542  L043.11  L045.11  L02..11  1542400  L044.11  L04z.00  L041z00  1542200  L042z00  L041z11  L045.00  L045z00  L011.00  L040011  L041100  L040.00  1542500  1542300  L010.11  L040900  1542Z00  L041y00  L044z00  L044z11  L045z11  1542700  L042y00  L040z00  L041w00  L045y11  1542100  L045y00  1542600  9535  L041000  L043z00  L044y00  L040y00  L040000  L044x00  L044y11  L042w00  L044x11  L02..12  L040500  L045000  L044100  L041500 | 198650006  13384007  43306002  307746006  307749004  59363009  286996009  10697004  2781009  198656000  13384007  307752007  184339009  43306002  34614007  34614007  161752007  184339009  2781009  16607004  2781009  307746006  59363009  16863000  16863000  198647008  198657009  198645000  16607004  307735008  2781009  307746006  59363009  16607004  184339009  413338003  10697004  58990004  16863000  16607004  161747002  17369002  44979007  156073000  307737000  62688006  156073000  450810001  198646004  17369002  198661003  16607004  156072005  307737000  156072005  307733001  198645000  307737000  18489003  198644001  198645000  156072005  198656000  17369002  59363009  161748007  198645000  7000009  198660002  198656000  199088001  161744009  35999006  307737000  59363009  307738005  35999006  307733001  156072005  275421004  198655001  8.65741E+14  73790007  307749004  307752007  198648003  48485000  609442008  67465009  156072005  176833006  307748007  161744009  35999006  161752007  307733001  307735008  307733001  307748007  737318003  198649006  156073000  59363009  34614007  156073000  161749004  307734007  307735008  59363009  161751000  59363009  161749004  156072005  275425008  161751000  9.06451E+14  156072005  307748007  17369002  198645000  198644001  34614007  161744009  199087006  198659007  198663000  156073000  307733001  59363009  16607004  176833006  307752007  17369002  307737000  30806007  307750004  198645000  17369002  161745005  59363009  59363009  161750004  156073000  307737000  17369002  156072005  307735008  737943000  307734007  237036002  17369002  17369002  161748007  16863000  184339009  307738005  275425008  307748007  176833006  67465009  275421004  609525000  307752007  156072005  307733001  307750004  161747002  85116003  161750004  59363009 | O02.0  O02.1  O03  O03.0  O03.1  O03.2  O03.3  O03.4  O03.5  O03.6  O03.7  O03.8  O03.9 |
| Hyperemesis Gravidarum | L13..11  L131000  L131200  L13..12  L130.00  L130000  L130z00  L130200  L131.00  L131z00  L130100  L131100 | 19569008  14094001  199028004  14094001  199025001  14094001  199025001  19569008  199023008  19569008  129598007  199022003  199027009  199025001  19569008  19569008  199025001  14094001 | O21.0  O21.1 |
| Ectopic pregnancy | L03..00  1544  584E.00  L03y200  L511.00  L03y000  L032.00  L03y300  L03y500  7E19000  L031200  7E13300  L031.00  7E13100  7E19011  L03y100  L03z.00  7E19012  L031000  L031z00  Lyu0000  L030.00  L03y.00  L031100  L03yz00  L030000 | 81130000  276881003  31601007  79586000  69532007  176928008  198624007  198617006  82661006  198617006  440537001  609525000  370382007  79586000  34801009  34801009  387617009  35656003  237253003  176928008  31601007  63596003  79586000  161763005  176929000  17433009  79290002  387616000  34801009  79586000  9899009  198620003  237253003  237254009  161763005  87605005  176929000  198626009  34801009  387615001  198620003  34801009  31601007  271415006  34801009  173300003, 176929000, 362811000000105, 906511000006107, 609525000  31601007 | O00  O00.0  O00.1  O00.2  O00.8. O36.7  O83.3, O00.9 |
| Gestational hypertension | L123200  L123.00  L123500  L123z00  L123600  L123100  L123000  L123400  L123300 | 198966006  48194001  237279007  237279007  237279007  237279007  198965005  198967002  307632004  48194001, 695711000000100, 40521000119100, 198968007  48194001, 695711000000100, 237279007  48194001 | O13 |
| Pre-eclampsia/Eclampsia | L124.12  L126300  L125.00  15A4.00  L12B.00  L124300  L126000  L125400  Q000.11, L124600, L124.00  L126.00  15A3.00  L124.11  L126100  L124500  L129.00, L12A.00, L126500  L125000  L125z00  L125100  L127100  L127.00  L126600  L127000  L124400  L124100  L127300  L124000  L124z00  L125200  L124200  L126z00  L125300  L126200  L126400  L127200, Lyu1.00, L127z00  L127400  15AB.00 | 1861661000006100, 398254007  161806007, 105651000119100, 237283007, 398254007, 41114007  46764007  41114007, 95605009, 237283007  46764007  15938005  46764007  237283007  15938005  15394000  95605009, 398254007, 648001000000103, 15938005  161807003  398254007  398254007  41114007  198992004  15394000  15938005  41114007  41114007  198992004  398254007  237283007  46764007  41114007  46764007  198983002  198997005  198991006  398254007, 398254007, 648001000000103, 398254007  41114007  237281009  398254007  15938005  198984008  198985009  95605009  237281009, 198609003, 198974007  15394000, 95605009, 198999008  198974007  398254007  198997005  161806007  198990007  198997005  15938005  199002002  198986005, 105651000119100,  161807003  46764007  398254007, 105651000119100, 15938005  46764007  268794002  199000005  199003007  41114007  398254007  398254007  95605009  198992004  198993009  398254007  46764007 | O11  O14.0  O14.1  O14.2,  O14.9,  O15.0  O15.1  O15.2,  O15.9,  O14  O15 |
| GDM | L180900  L180811  L180.00  L180300  ZC2CB00  L180800  L180100  L180z00  L180000  ZV13F00 | 276560009, 46894009  46894009  75022004, 11687002  199223000  199223000, 76751001, 11687002  199223000  472971004  11687002  199223000  702849009  472971004, 40791000119105, 816261000000105, 40801000119106, 40791000119105, 721151003  199225007  199227004  237627000, 75022004, 11687002  439051004  11687002  11687002  11687002, 40801000119106 | O24  O24.4, O24.9 |

|  |  |  |  |
| --- | --- | --- | --- |
| Anxiety | E200.00  E200300  E200100  Eu41012  Eu41200  Z522400  Eu41111  E202B00  E202800  E200400  E202000  E202C00  Eu40000  E202100  E200z00  E200500  E200200  Eu41.00  E292000  E200000  Eu40z11  Eu41211  Z4L1.00  Eu41000  Eu60600  8G94.00  Eu40.00  Eu40200  E202.12  Eu41100  Eu40213  Eu40100  Eu40300  Eu40214  E202200  2258  Eu40012  Eu34114  Eu40011  Eu51511  Eu93000  Eu40212, E202300, Eu40011  Eu51511  Eu93000  Eu40212, E202500, Eu41y00  Eu93100  Eu41z11  Z481.00  Z4I7211  Eu40y00  E202600  Eu41y11  8HHp.00  E202700  Eu93200  E2D0.00, E202400  Eu40z00, E2D0z00  Eu41300  Eu41113  Eu93y12  Eu45215  Eu40211  1B13.12  8IH5300 | 228560001  386810004  313087008  54587008  313087008  191723004, 1818111000006100, 313087008  162723006, 853241000006103, 17496003  191733007  17496003  48694002  231501003  38617005  64165008  191722009  386810004  313087008  21897009, 1861181000006100, 54307006  371631005  231504006  48694002  192108001, 1037451000000100, 386810004  47372000  21897009, 972931000006101, 191709001  386810004  25501002  231504006  54587008  11806006, 191725006, 197480006  54587008  192108001  21897009  47372000, 851141000006108, 18193002  191722009  231528008  17496003  54587008  58963008  25501002, 933461000000100, 191723004, 563201000000101, 197480006  198288003  191722009  198288003  386808001  231504006  386810004  371631005  11806006, 199101000000102, 54307006, 191724005, 197480006  231504006  56576003  13438001  70691001  198288003  21897009  371631005  231504006  198288003  19887002  247854002  34563004  61569007  231504006  192611004  419145002, 1037471000000100, 79015004  191708009, 197480006, 162723006  197480006  386810004  54307006, 909691000006109, 710060004, 191726007, 192611004  197480006  21897009  70691001  371631005  207363009  228560001, 386810004, 563201000000101, 70691001  48694002, 851351000006108, 54587008  54587008  13438001  25501002 | F06.4  F40  F40.0  F40.1  F40.2  F40.8, F40.9, F41  F41.0  F41.1  F41.2  F41.3  F41.8, F41.9, F60.6  F93.0  F93.1  F93.2 |
| **Depression** | E2B..00  Eu32z11  E200300  E204.00  E204.11  E2B1.00  Eu41200, Eu25111, 9kQ..00  E112.14  E135.00  E290.00  E11..12  62T1.00, Eu32z00, E2B0.00  Eu32z12  Eu33.00  Eu32.00  Eu53012  E112.11  Eu32z14  E113700  E112.12  Eu32y00  E113.11  E112.13  E112z00  Eu32.13  Eu34113  Eu41211  Eu34100  E130.00  Eu34111  Eu33.15  Eu33.11  Eu33.13  Eu32.11  E11z200  Eu32100  Eu32200  E112.00  Eu32400  Eu32y11  E118.00, Eu25100  Eu33212, Eu33211  Eu32000  Eu32300  9H91.00  9H90.00  Eu53011, E113200, E113.00, E112200, E112300, Eu34114, E112100, E291.00  Eu33315  E130.11  Eu32.12  6G00.00  Eu3y111  212S.00  Eu33.12  Eu20400  E002100  Eu32212  Eu33311  Eu32313  Eu32311, E113400, E113z00, E113300, E11y200  E001300  Eu32z13  Eu33.14  Eu32314, E113100  Eu33100, R007z13, Eu33000, 9H92.00  8CAa.00  9k4..00  Eu33314, E112400, 8HHq.00  Eu92000  Eu33313  Eu33200, E112000  E113000, E290z00  Eu33z11  Eu33316, Eu25112, E002z00  Eu32211  9HA0.00  E004300, Eu33z00, E002.00  8BK0.00  E02y300  Eu33300  Eu33y00  9Ov..00  Eu32312  Eu32y12  Eu32213  9k40.00  9Ov0.00  9Ov1.00, Eu33214, 9Ov4.00  9Ov3.00  9Ov2.00, Eu32600  Eu32500  Eu32700  Eu32800, Eu32B00  8IH5200, Eu32900  Eu32A00 | 1771531000006100, 1972451000006100, 1972541000006100, 1972541000006100, 1972911000006100, 1973381000006100, 1975211000006100, 1975261000006100, 1975321000006100, 1976921000006100, 310495003  395072006  191613003  82218004  401174001  15639000, 1086471000000100, 15639000, 609311000000100, 84760002, 430421000000104, 310497006, 784051000000106, 397701000000102, 191495003, 251000119105, 1975231000006100, 15639000, 36923009, 395072006, 79298009, 465441000000108, 231500002  192080009  413974004  161469008  310496002, 609311000000100, 87414006  268621008, 15639000  191611001, 191676002  310497006, 1976491000006100, 268621008, 191659001, 361761000000106, 231504006, 35489007, 36923009, 609311000000100, 413972000  310497006, 310496002, 247803002, 191613003, 191616006, 192049004, 909681000006106, 166481000000107, 199111000000100, 1038261000000100, 87414006  87414006  231542000, 84760002, 1976021000006100, 191676002  36474008, 35489007  87414006  35489007  83458005  310495003  231504006, 1976231000006100, 73867007, 58703003, 191610000  84760002, 1110911000000100, 191616006  191604000  310497006  191457008  247803002, 1976251000006100, 35489007, 784051000000106, 1975191000006100, 1972071000006100, 1973551000006100, 1972771000006100, , 196381000000100, 161469008, 231499006  83458005  35489007  310496002  247803002  268621008, 764611000000100, 401174001, 75084000, 430421000000104, 191457008  78667006  191616006, 790961000000101, 1976051000006100, 231485007  35489007, 933441000000101, 1975981000006100, 1972111000006100, 161469008  231542000, 1972201000006100, 395072006, 717211000000107, 35489007  231504006  300706003, 465441000000108, 395072006, 908731000006105, 191459006  191616006  28475009  58703003  231504006, 853871000006107, 73867007, 1975991000006100, 361761000000106, 231485007  35489007  58703003  231504006  274948002  413169006  231499006  191604000, 79298009, 716421000000103, 87414006  36923009  87414006  73867007, 166291000000108, 84760002, 191613003  28475009  40568001, 1057351000000100, 191466007  82218004, 1976271000006100, 191676002, 310497006  36923009, 1972661000006100, 87414006  191616006  191616006, 713831000000108, 716681000000100, 716961000000102, 78667006  35489007, 310495003, 191616006  28475009  192046006, 87512008, 191604000  191455000  40568001, 1823881000006100, 231500002, 1976411000006100, 1976211000006100, 923921000000104, 413973005  310495003  300706003  415044007  78667006  58703003, 36923009, 191676002, 251000119105, 191604000, 939961000006100, 268621008, 717261000000109, 397701000000102, 191613003  192079006  191659001, 1086471000000100, 191495003  310497006, 1972311000006100, 1972131000006100 | F20.4, F25.1, F32  F32.0  F32.1  F32.2  F32.3  F32.8, F32.9, F33, F33.0  F33.1  F33.2  F33.3, F33.8, F33.9, F34.1  F41.2  F92.0 |
| Preterm Birth | Calculate from the gestational age variable in the maternity tail  Gestational age<37weeks | | \| O60 \| \| --- \| \| O60.1 \| \| O60.2 \| \| O60.3 \| \| P07.2 \| \| P07.3 \| \| P59.0 \| \| P61.2 \| |
| Caesarean section | Calculated from Delivery method variable in maternity tail and OPCS coded in HES linked data  ICD10 Codes   \| O82 \| \| --- \| \| O82.0 \| \| O82.1 \| \| O82.2 \| \| O82.8 \| \| O82.9 \| | | \| **OPCS Codes**  R17 \| \| --- \| \| R17.1 \| \| R17.2 \| \| R17.8 \| \| R17.9 \| \| R18 \| \| R18.1 \| \| R18.2 \| \| R18.8 \| \| R18.9 \| \| R19 \| \| R19.1 \| \| R19.8 \| \| R19.9 \| \| R20 \| \| R20.1 \| \| R20.2 \| \| R20.8 \| \| R20.9 \| \| R21 \| \| R21.1 \| \| R21.2 \| \| R21.3 \| \| R21.4 \| \| R21.5 \| \| R21.8 \| \| R21.9 \| \| R22 \| \| R22.1 \| \| R22.2 \| \| R22.3 \| \| R22.8 \| \| R22.9 \| \| R23 \| \| R23.1 \| \| R23.2 \| \| R23.8 \| \| R23.9 \| \| R24 \| \| R24.9 \| \| R25 \| \| R25.1 \| \| R25.2 \| \| R25.8 \| \| R25.9 \| \| R27 \| |

**
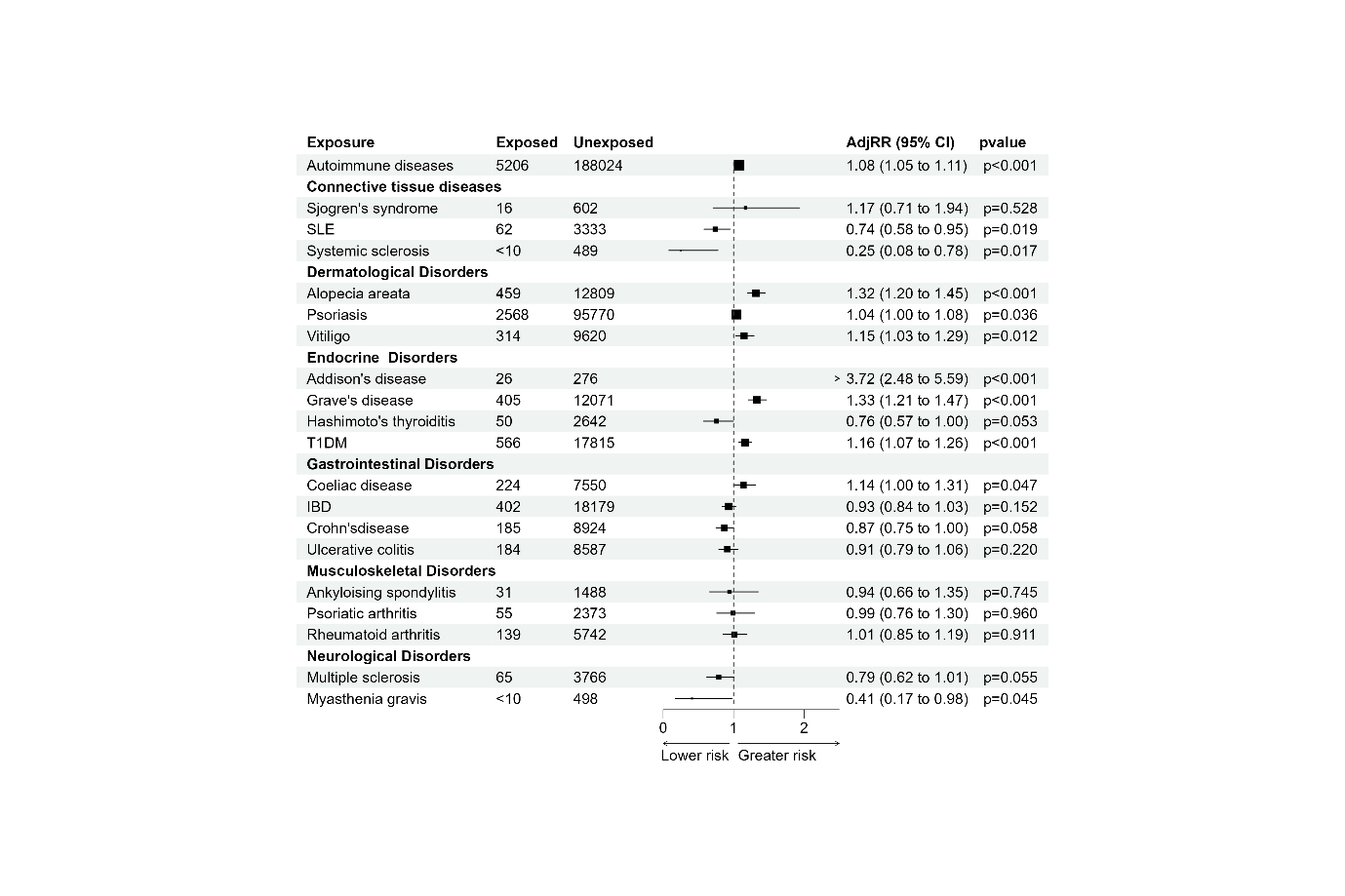
**

Exposed=No. of events in exposed, Unexposed=No. of events in Unexposed, IBD=Inflammatory bowel disease, T1DM=Type 1 diabetes mellitus, AdjRR=Adjusted Risk ratio

# Figure 5 Forest plot describing association of autoimmune diseases in women and Hyperemesis gravidarum


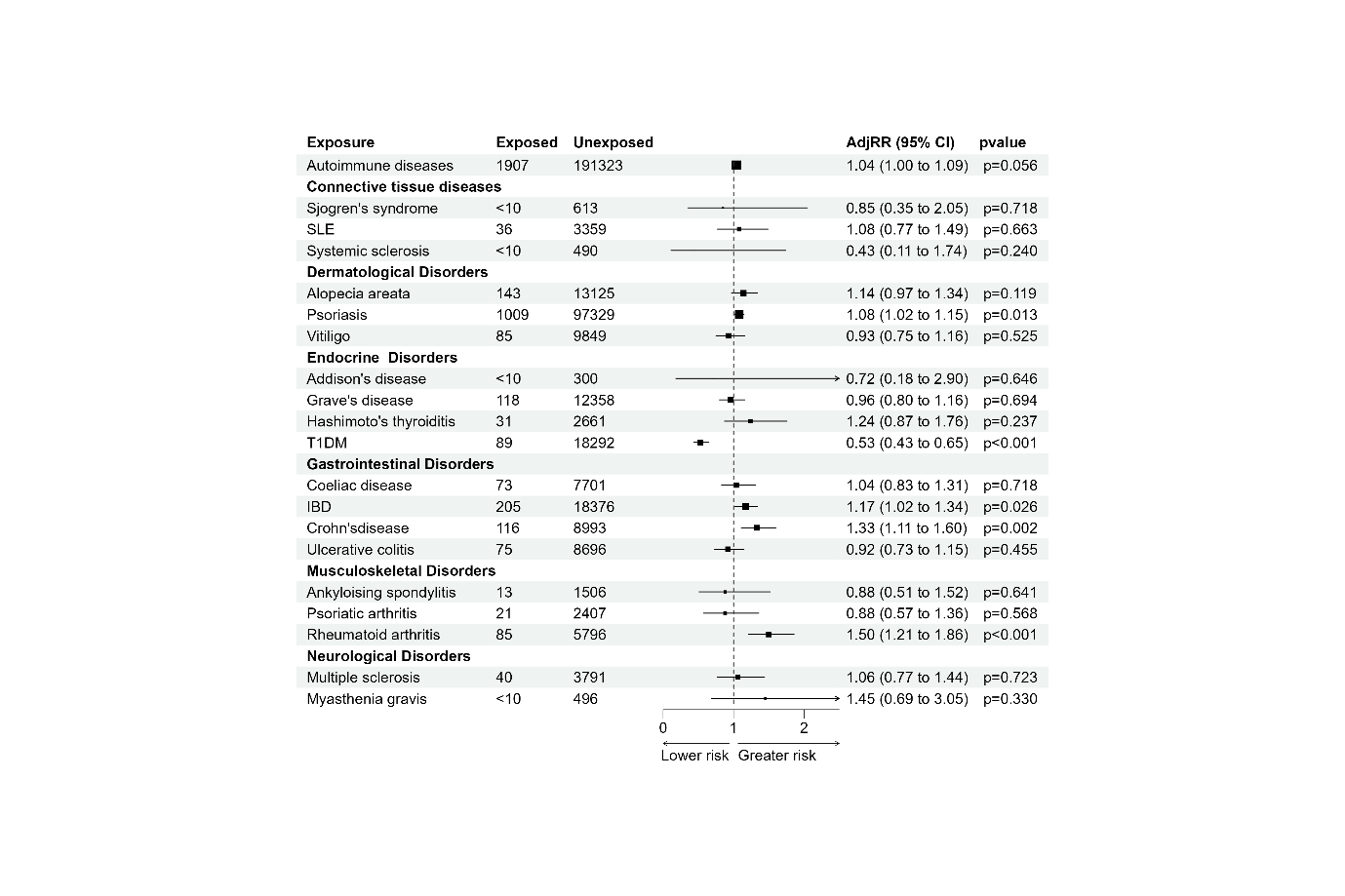


Exposed=No. of events in exposed, Unexposed=No. of events in Unexposed, IBD=Inflammatory bowel disease, T1DM=Type 1 diabetes mellitus, AdjRR=Adjusted Risk ratios

# Figure 6 Forest plot describing association of autoimmune diseases in women and Ectopic pregnancy


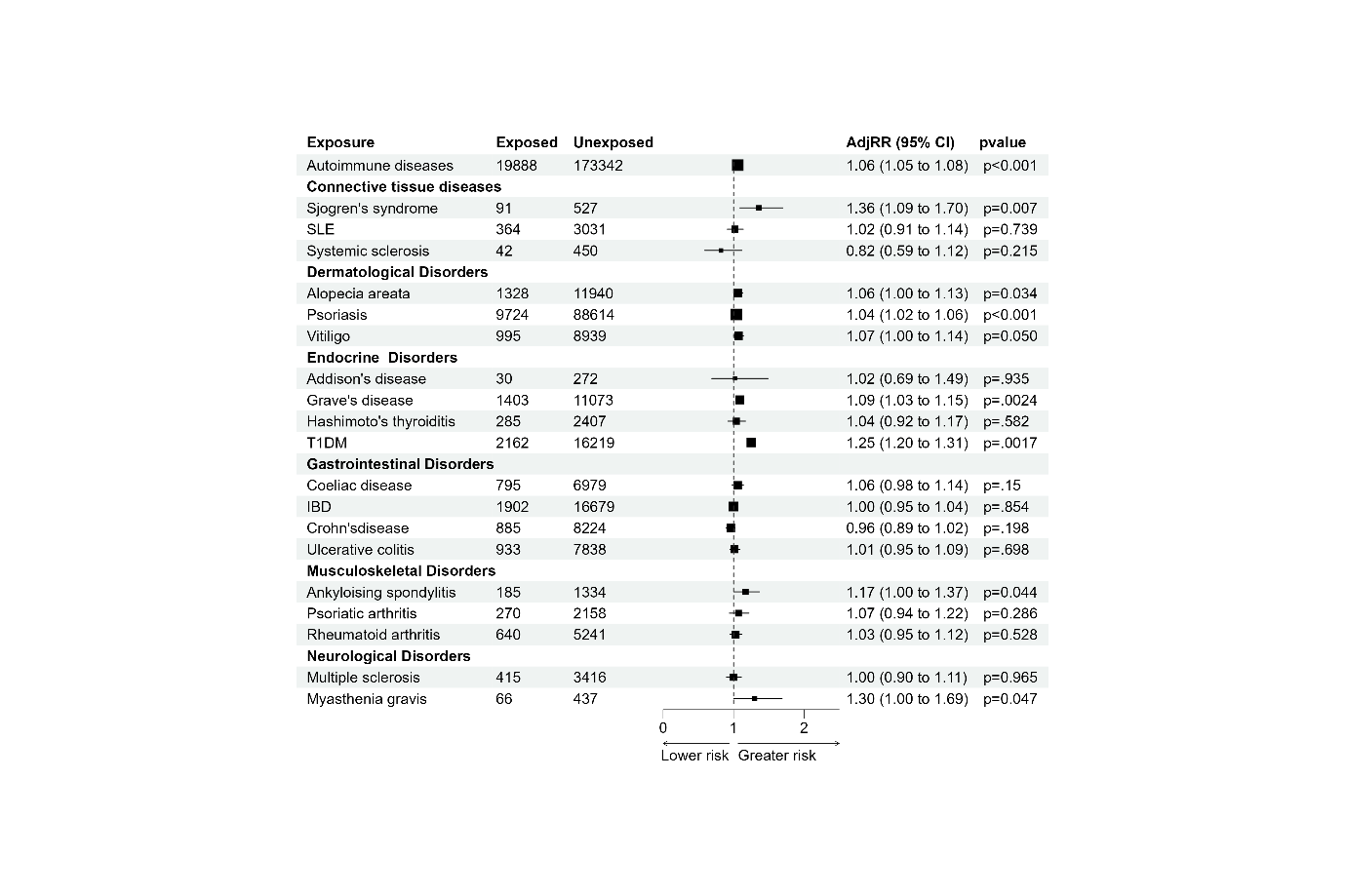


Exposed=No. of events in exposed, Unexposed=No. of events in Unexposed, IBD=Inflammatory bowel disease, T1DM=Type 1 diabetes mellitus, AdjRR=Adjusted Risk ratios

# Figure 7 Forest plot describing association of autoimmune diseases in women and Miscarriage


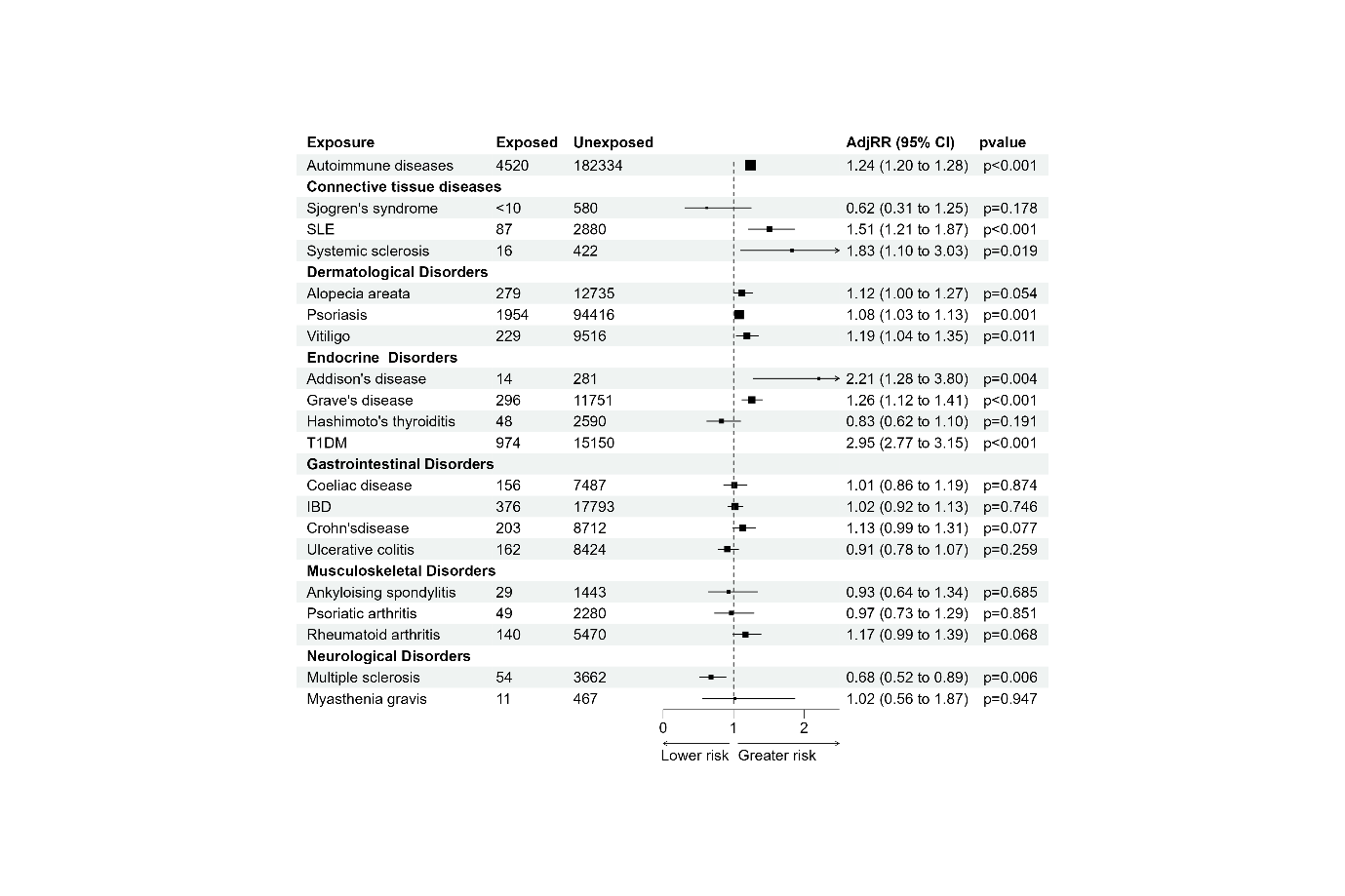


Exposed=No. of events in exposed, Unexposed=No. of events in Unexposed, IBD=Inflammatory bowel disease, T1DM=Type 1 diabetes mellitus, AdjRR=Adjusted Risk ratios

# Figure 8 Forest plot describing association of autoimmune diseases in women and Gestational hypertension


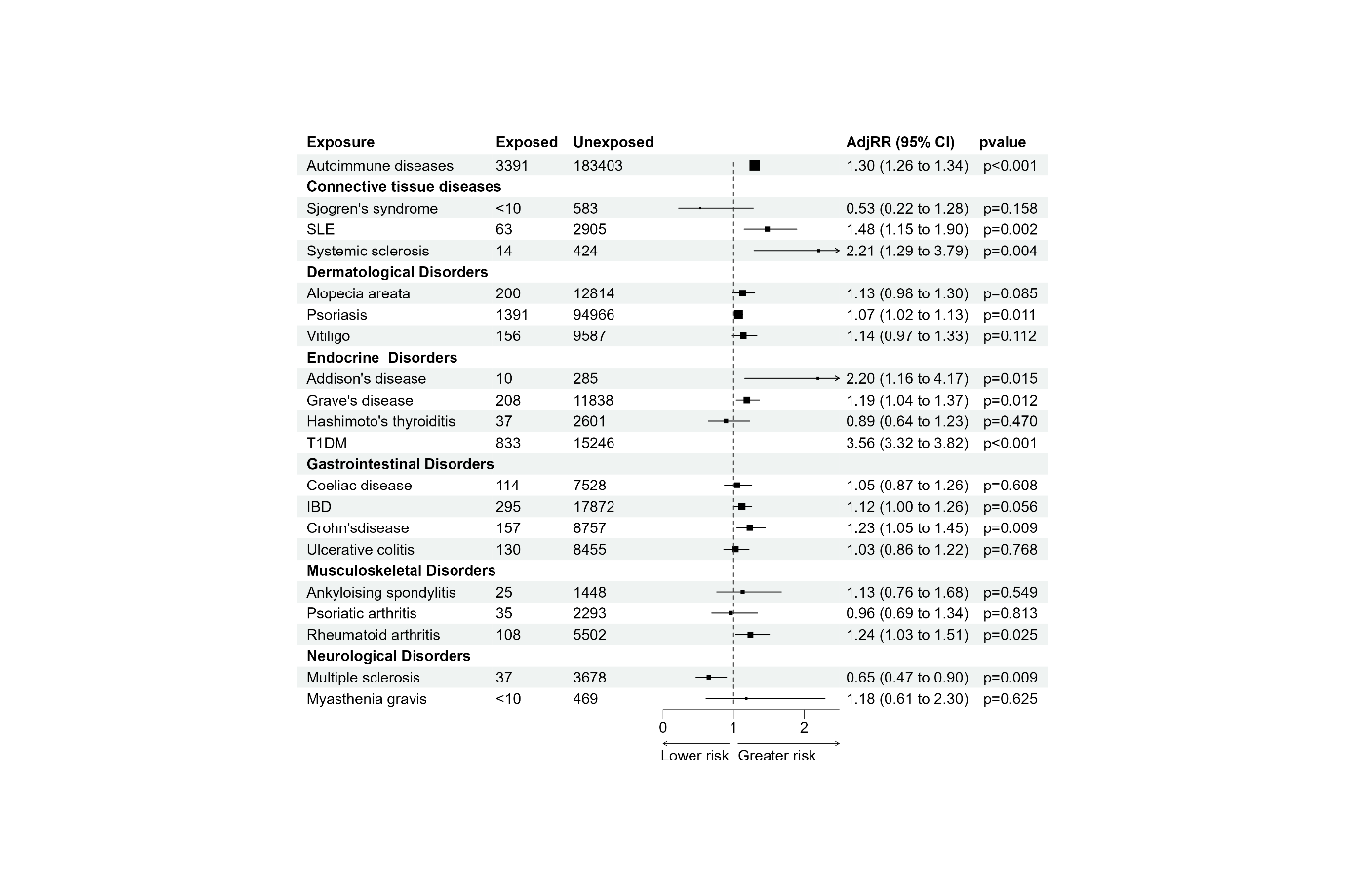


Exposed=No. of events in exposed, Unexposed=No. of events in Unexposed, IBD=Inflammatory bowel disease, T1DM=Type 1 diabetes mellitus, AdjRR=Adjusted Risk ratios

# Figure 9 Forest plot describing association of autoimmune diseases in women and Pre-eclampsia


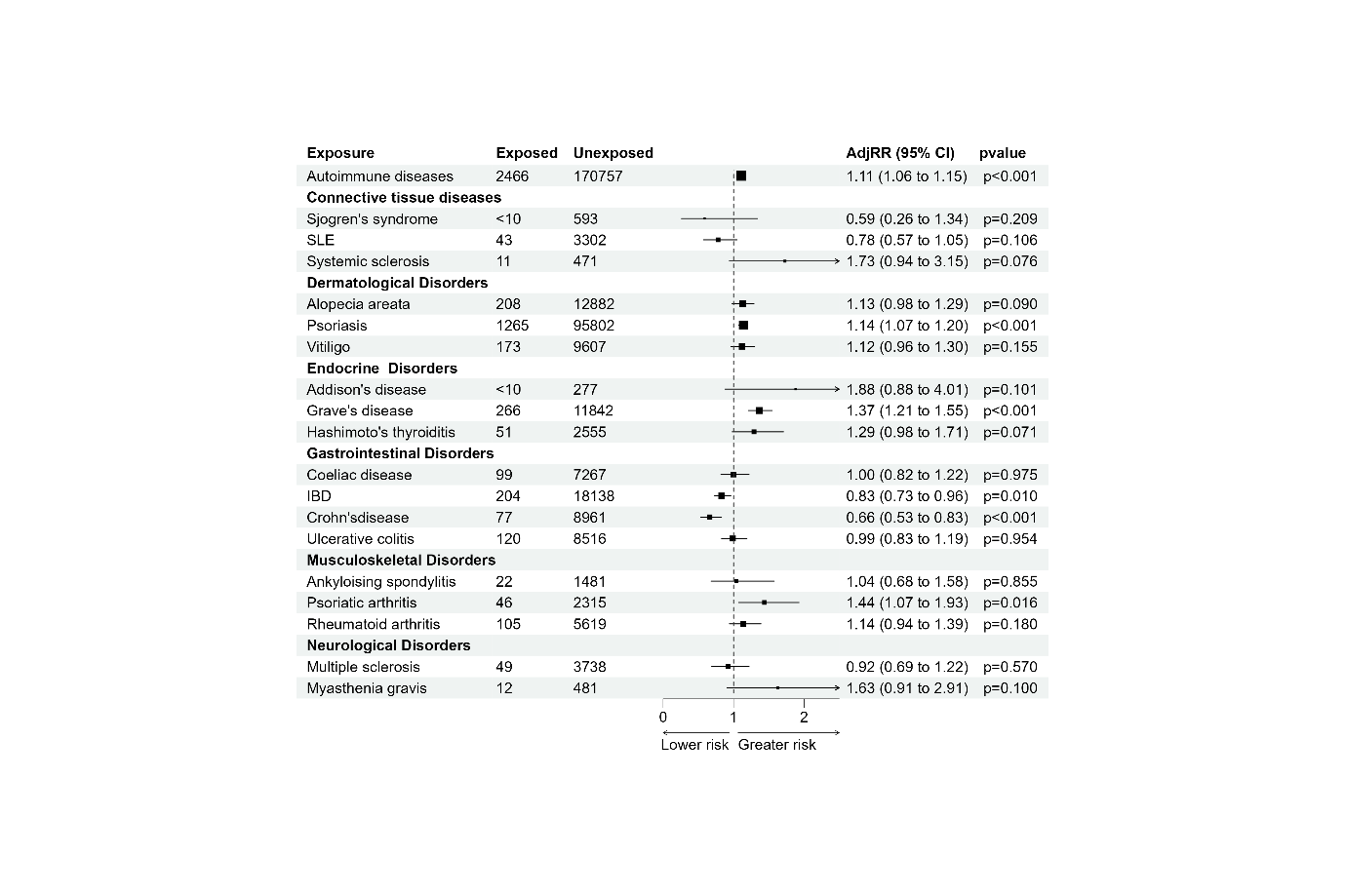


Exposed=No. of events in exposed, Unexposed=No. of events in Unexposed, IBD=Inflammatory bowel disease, T1DM=Type 1 diabetes mellitus, AdjRR=Adjusted Risk ratios

# Figure 10 Forest plot describing association of autoimmune diseases in women and Gestational diabetes mellitus


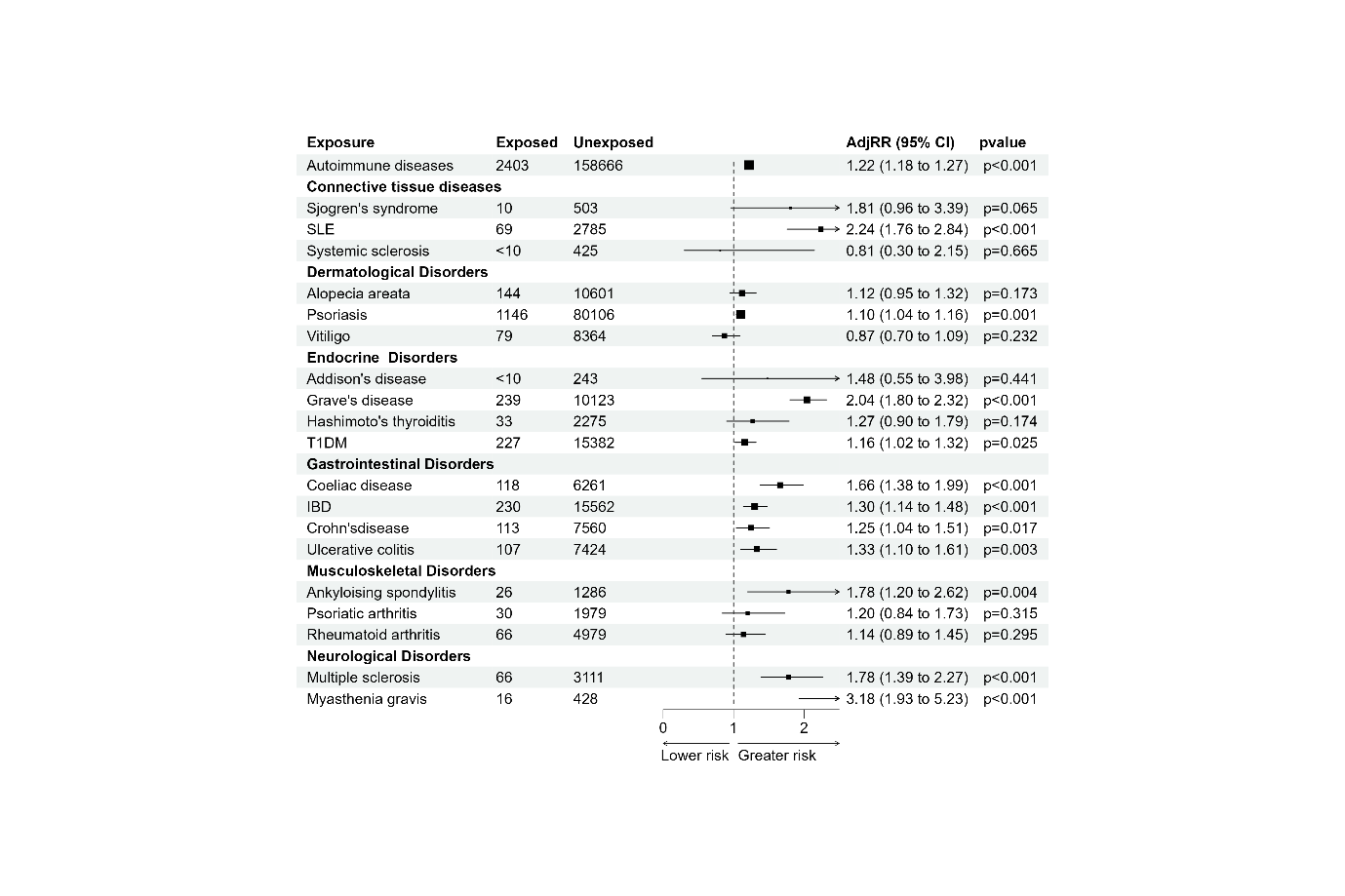


Exposed=No. of events in exposed, Unexposed=No. of events in Unexposed, IBD=Inflammatory bowel disease, T1DM=Type 1 diabetes mellitus, AdjRR=Adjusted Risk ratios

# Figure 11 Forest plot describing association of autoimmune diseases in women and Antenatal anxiety


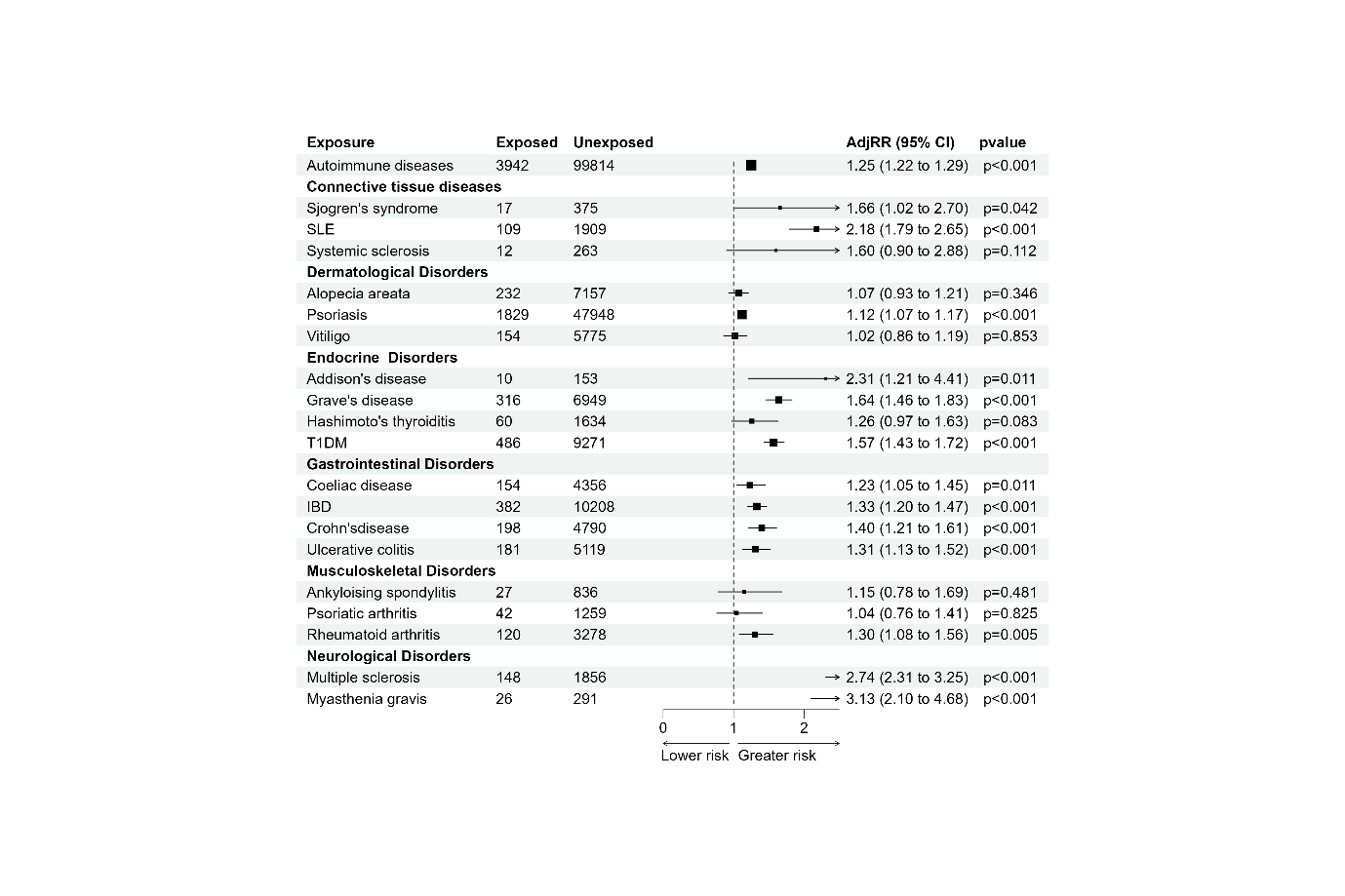


Exposed=No. of events in exposed, Unexposed=No. of events in Unexposed, IBD=Inflammatory bowel disease, T1DM=Type 1 diabetes mellitus, AdjRR=Adjusted Risk ratios

# Figure 12 Forest plot describing association of autoimmune diseases in women and Antenatal depression


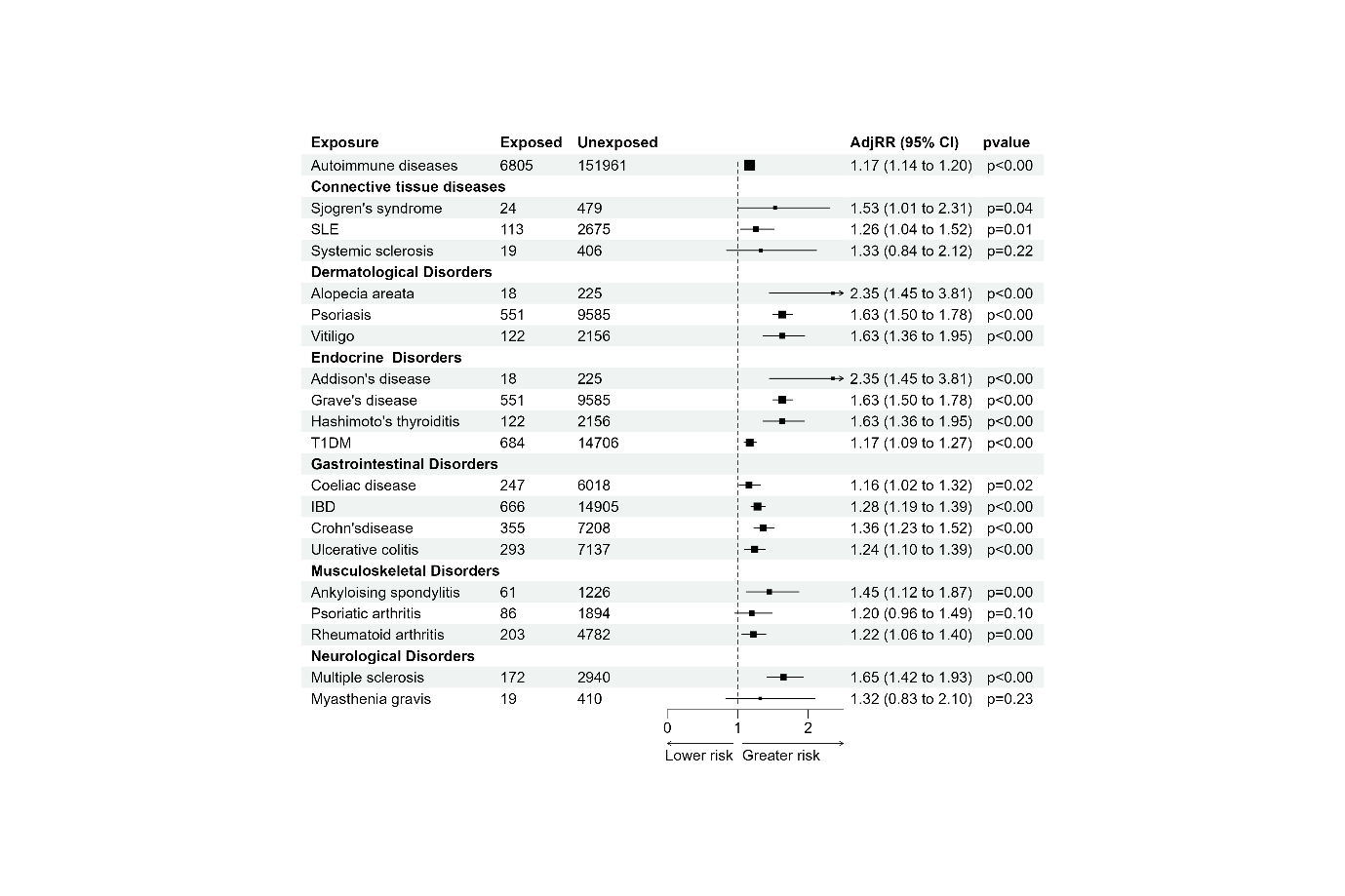


Exposed=No. of events in exposed, Unexposed=No. of events in Unexposed, IBD=Inflammatory bowel disease, T1DM=Type 1 diabetes mellitus, AdjRR=Adjusted Risk ratios

# Figure 13 Forest plot describing association of autoimmune diseases in women and postnatal anxiety


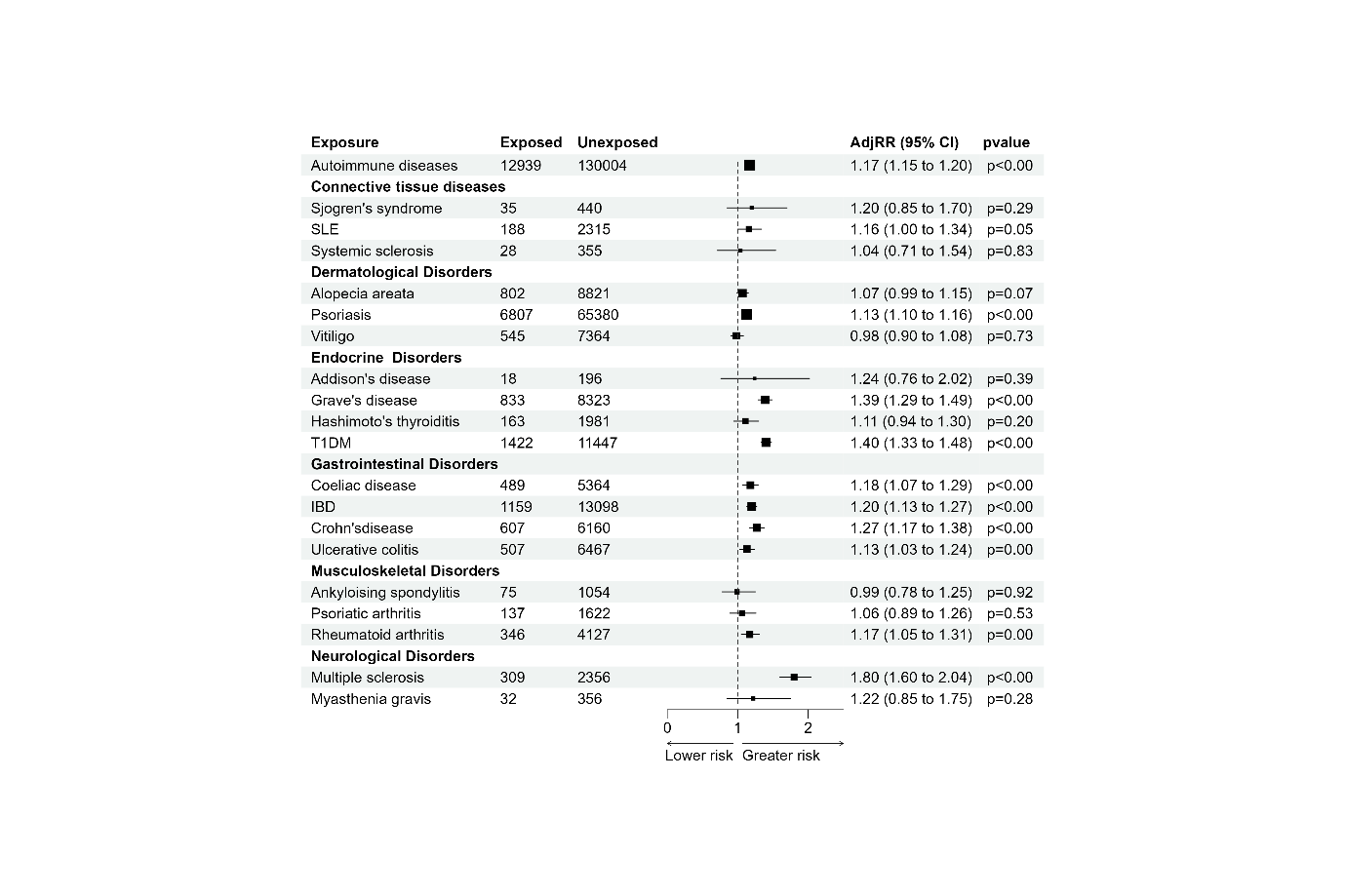


Exposed=No. of events in exposed, Unexposed=No. of events in Unexposed, IBD=Inflammatory bowel disease, T1DM=Type 1 diabetes mellitus, AdjRR=Adjusted Risk ratios

# Figure 14 Forest plot describing association of autoimmune diseases in women and postnatal depression


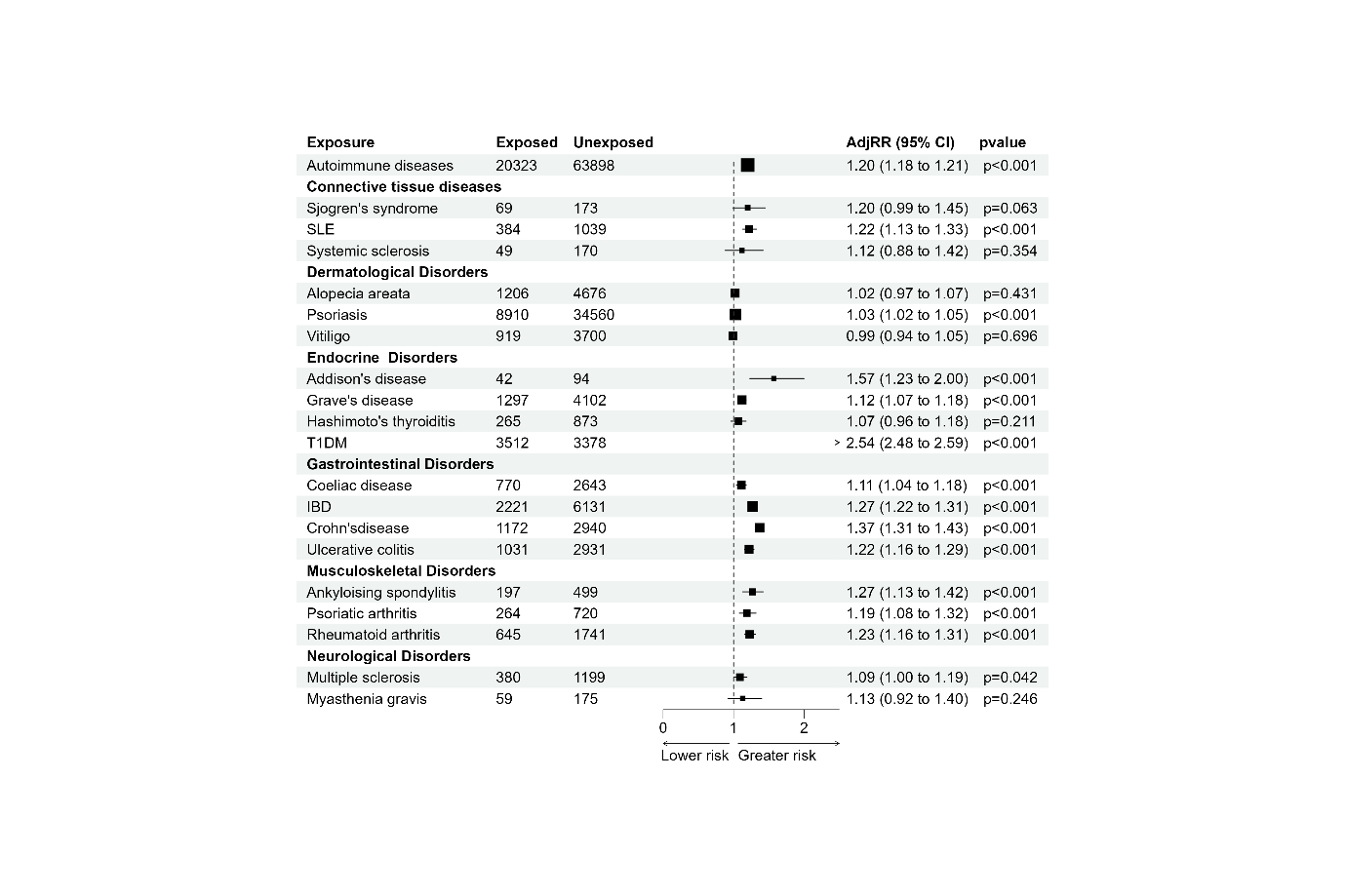


Exposed=No. of events in exposed, Unexposed=No. of events in Unexposed, IBD=Inflammatory bowel disease, T1DM=Type 1 diabetes mellitus, AdjRR=Adjusted Risk ratios

# Figure 15 Forest plot describing association of autoimmune diseases in women and Caesarean section


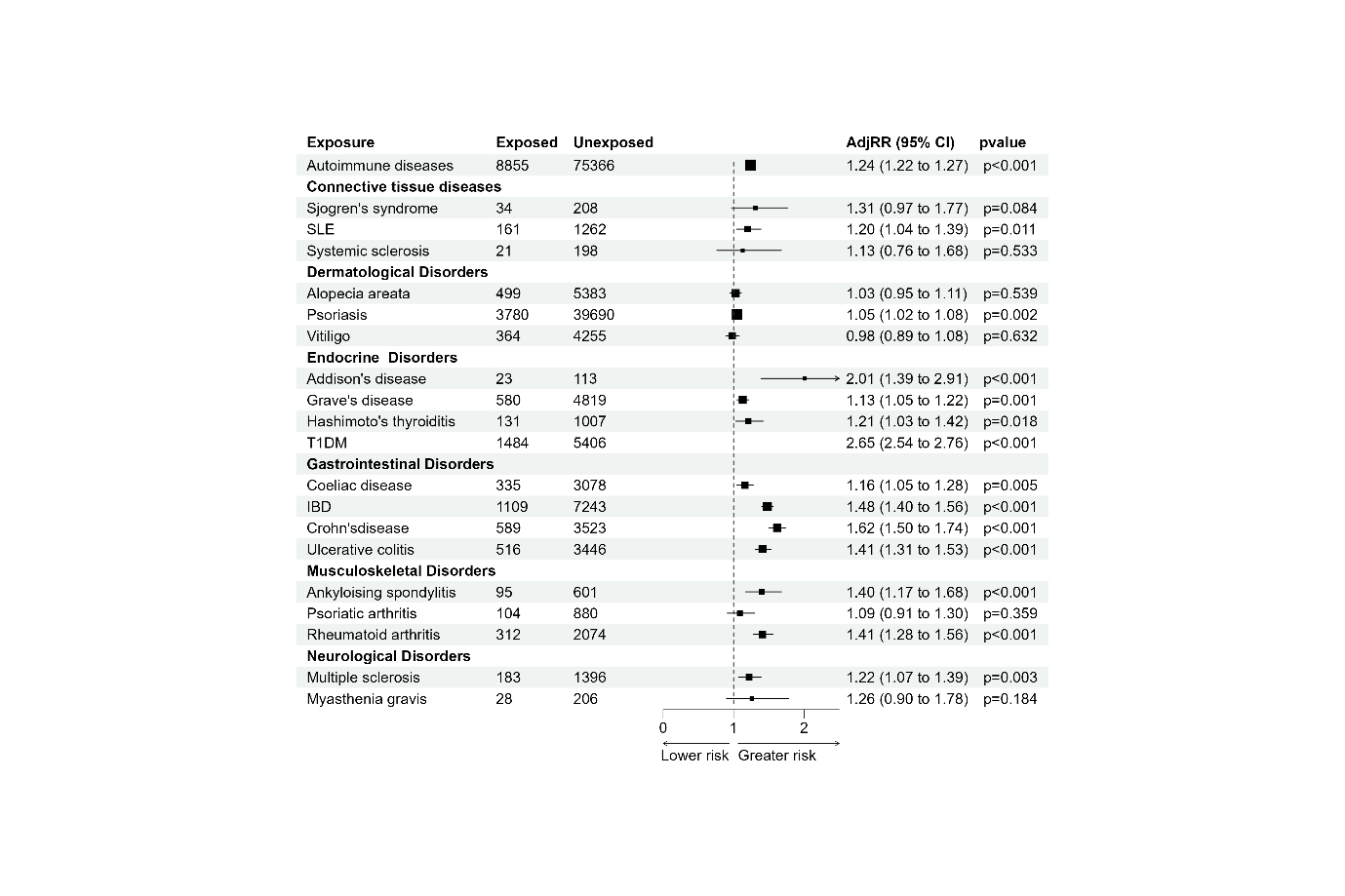


Exposed=No. of events in exposed, Unexposed=No. of events in Unexposed, IBD=Inflammatory bowel disease, T1DM=Type 1 diabetes mellitus, AdjRR=Adjusted Risk ratios

# Figure 16 Forest plot describing association of autoimmune diseases in women and Elective Caesarean section


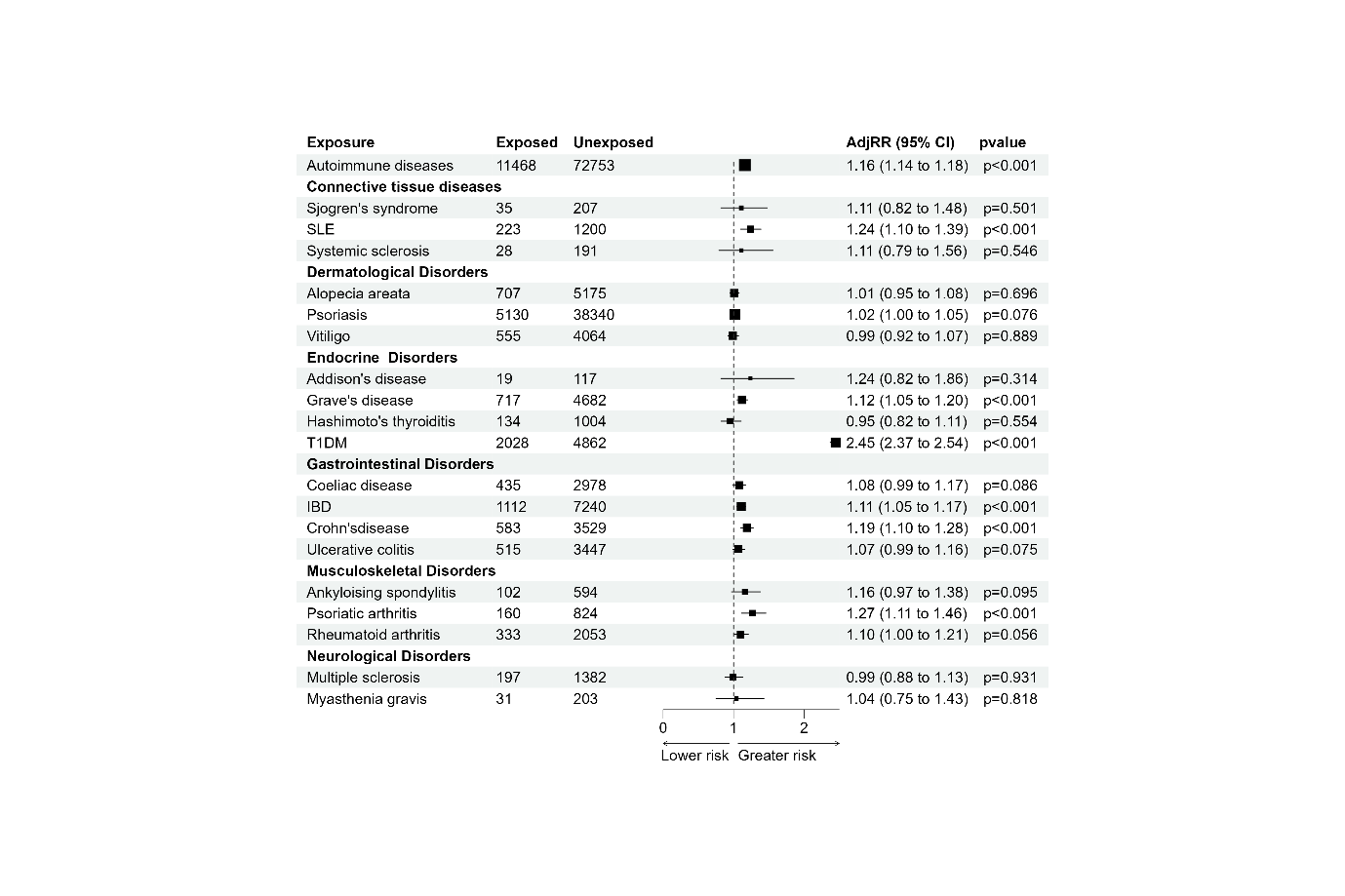


Exposed=No. of events in exposed, Unexposed=No. of events in Unexposed, IBD=Inflammatory bowel disease, T1DM=Type 1 diabetes mellitus, AdjRR=Adjusted Risk ratios

# Figure 17 Forest plot describing association of autoimmune diseases in women and Emergency Caesarean section


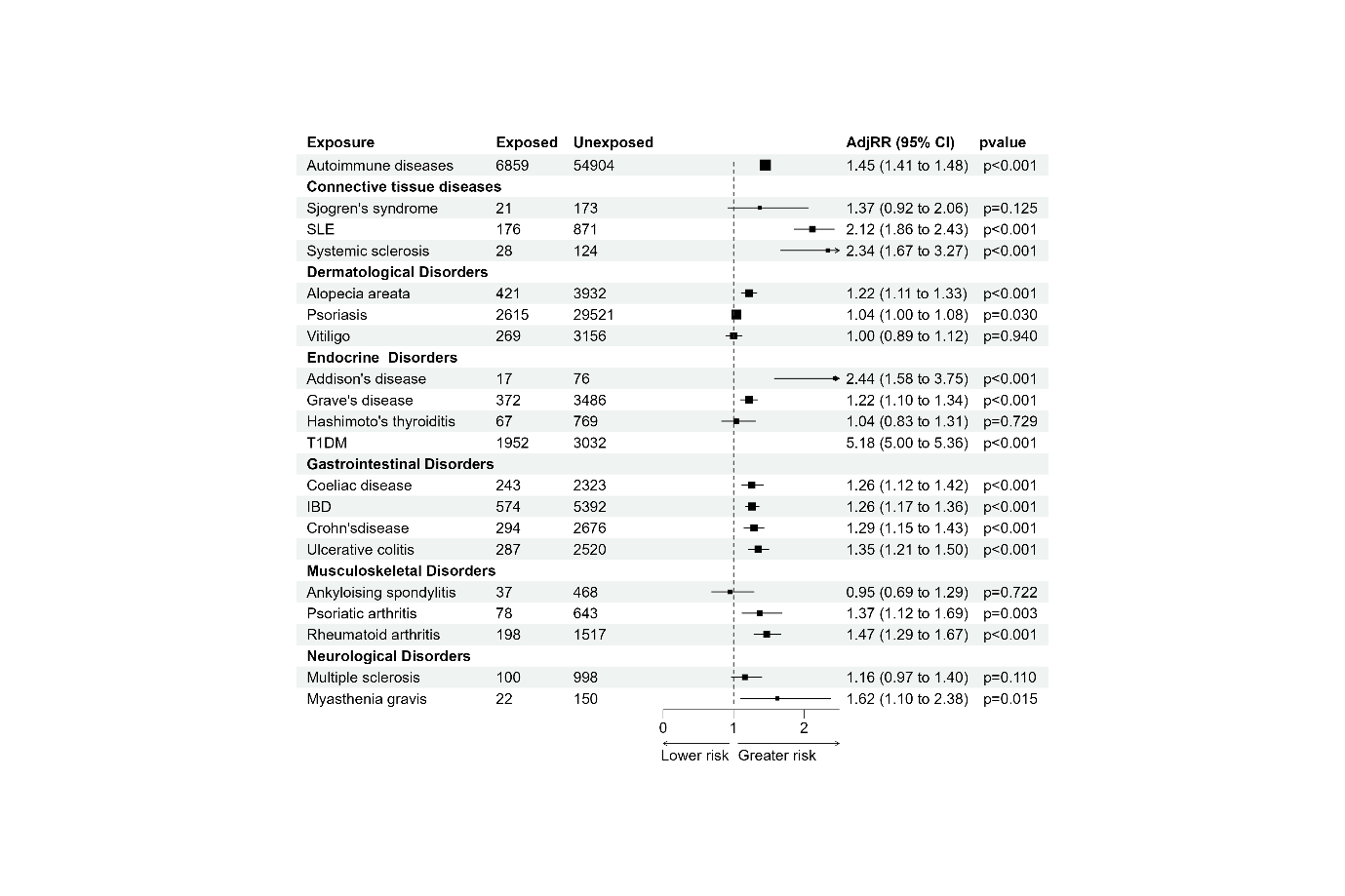


Exposed=No. of events in exposed, Unexposed=No. of events in Unexposed, IBD=Inflammatory bowel disease, T1DM=Type 1 diabetes mellitus, AdjRR=Adjusted Risk ratios

# Figure 18 Forest plot describing association of autoimmune diseases in women and Preterm birth


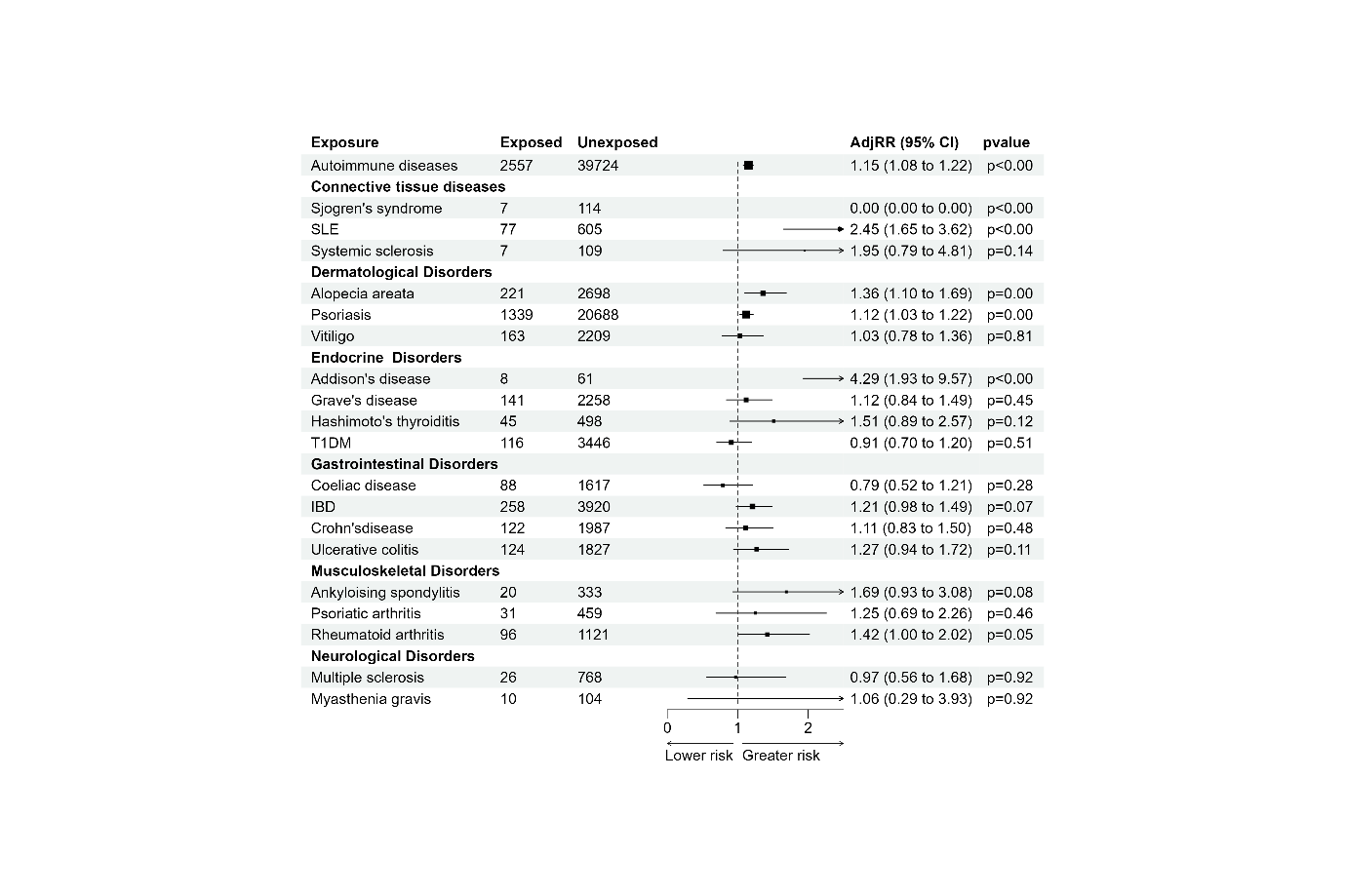


Exposed=No. of events in exposed, Unexposed=No. of events in Unexposed, IBD=Inflammatory bowel disease, T1DM=Type 1 diabetes mellitus, AdjRR=Adjusted Risk ratios

# Figure 19 Forest plot describing association of autoimmune diseases in women and Small for gestational age


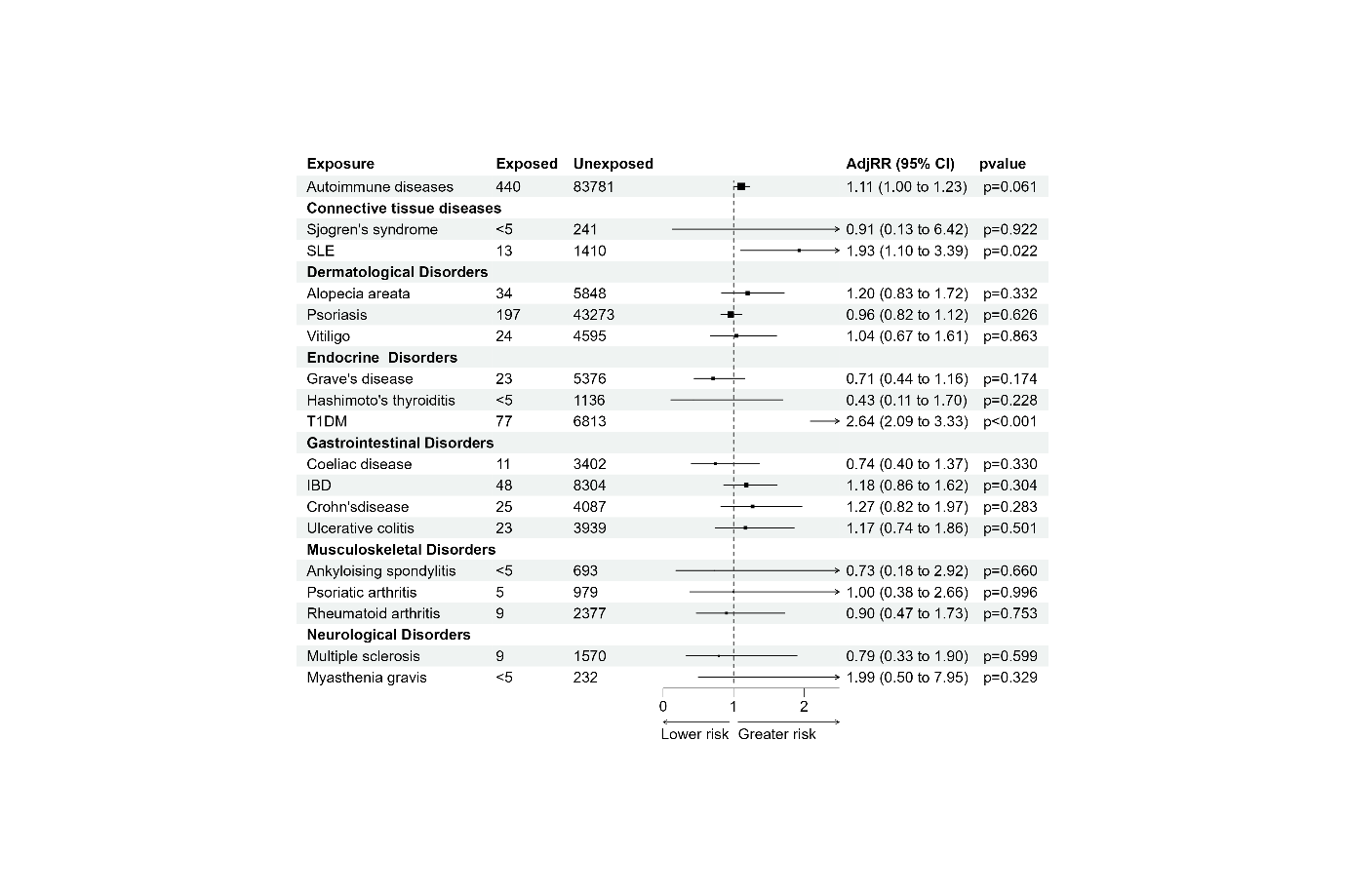


Exposed=No. of events in exposed, Unexposed=No. of events in Unexposed, IBD=Inflammatory bowel disease, T1DM=Type 1 diabetes mellitus, AdjRR=Adjusted Risk ratios

# Figure 20 Forest plot describing association of autoimmune diseases in women and Stillbirth


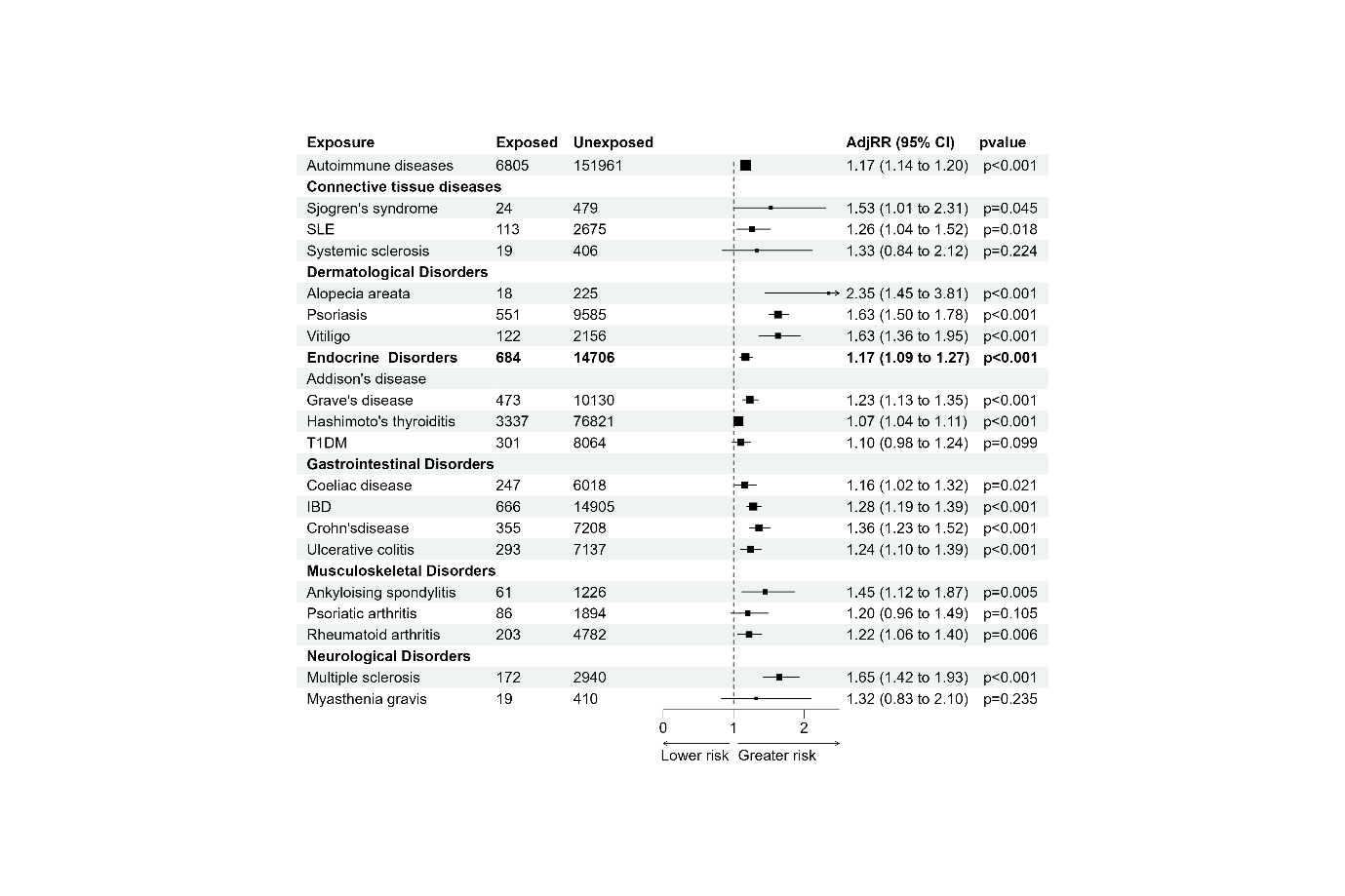


Exposed=No. of events in exposed, Unexposed=No. of events in Unexposed, IBD=Inflammatory bowel disease, T1DM=Type 1 diabetes mellitus, AdjRR=Adjusted Risk ratios

# Figure 21 Forest plot describing association of autoimmune diseases in women and Perinatal anxiety


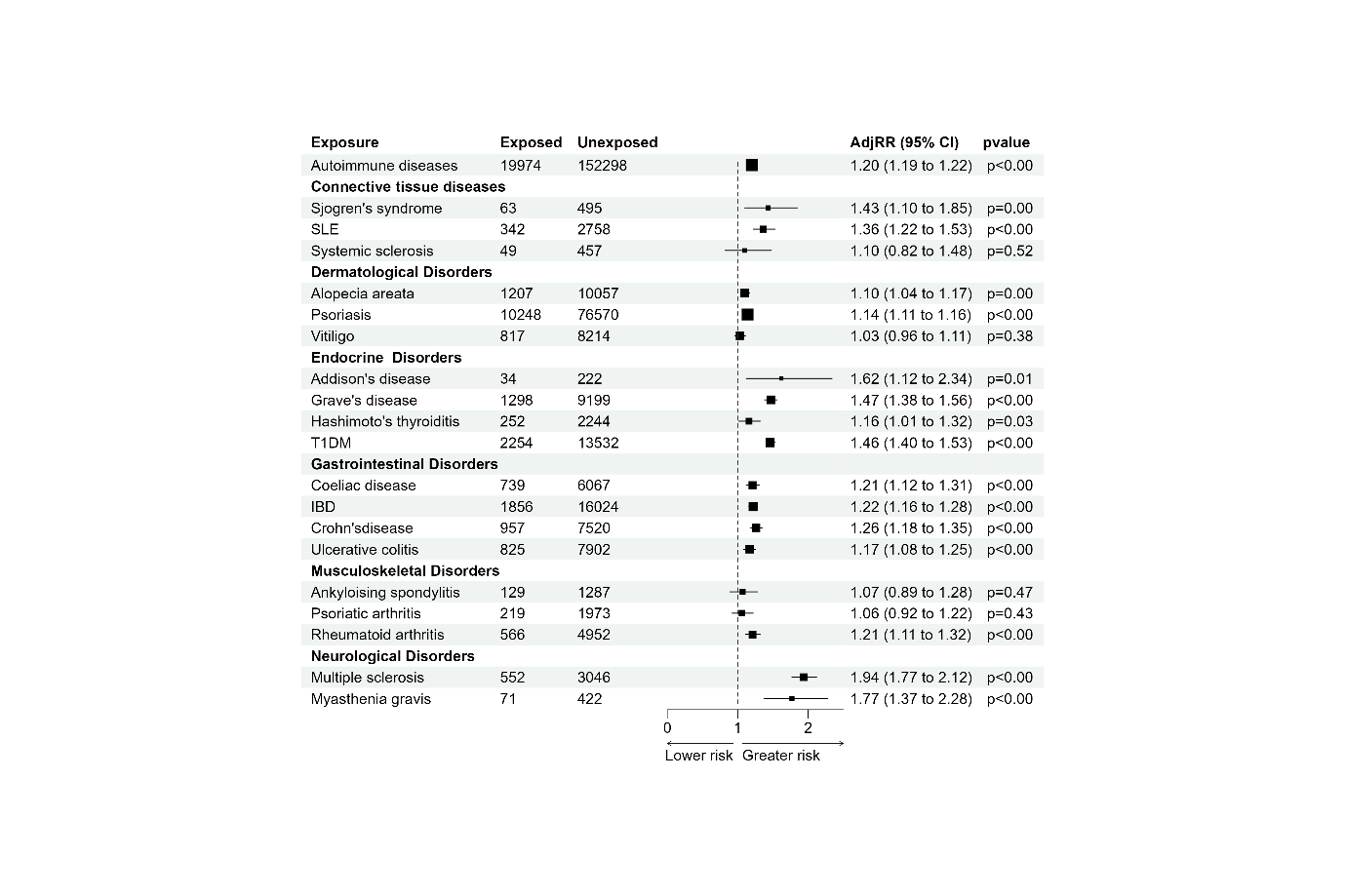


Exposed=No. of events in exposed, Unexposed=No. of events in Unexposed, IBD=Inflammatory bowel disease, T1DM=Type 1 diabetes mellitus, AdjRR=Adjusted Risk ratios

# Figure 22 Forest plot describing association of autoimmune diseases in women and Perinatal depression

# Table 17 Heat Map-diseases and adverse pregnancy outcomes

|  | **Hyperemesis Gravidarum** | **Ectopic pregnancy** | **Miscarriage** | **Gestational**  **hypertension** | **Pre-eclampsia**  **/eclampsia** | **GDM** | **PTB** | **SGA** | **CS** | **Stillbirth** | **Anxiety** | **Depression** |
| --- | --- | --- | --- | --- | --- | --- | --- | --- | --- | --- | --- | --- |
| Any AID |  |  |  |  |  |  |  |  |  |  |  |  |
|  | | | | | | | | | | | | |
| Sjogren’s S |  |  |  |  |  |  |  |  |  |  |  |  |
| SLE |  |  |  |  |  |  |  |  |  |  |  |  |
| Systemic S |  |  |  |  |  |  |  |  |  |  |  |  |
|  | | | | | | | | | | | | |
| Alopecia A |  |  |  |  |  |  |  |  |  |  |  |  |
| Psoriasis |  |  |  |  |  |  |  |  |  |  |  |  |
| Vitiligo |  |  |  |  |  |  |  |  |  |  |  |  |
|  | | | | | | | | | | | | |
| Addison Ds |  |  |  |  |  |  |  |  |  |  |  |  |
| Graves’ Ds |  |  |  |  |  |  |  |  |  |  |  |  |
| Hashimoto D |  |  |  |  |  |  |  |  |  |  |  |  |
| T1DM |  |  |  |  |  |  |  |  |  |  |  |  |
|  | | | | | | | | | | | | |
| Coeliac Ds |  |  |  |  |  |  |  |  |  |  |  |  |
| IBD |  |  |  |  |  |  |  |  |  |  |  |  |
| Crohn Ds |  |  |  |  |  |  |  |  |  |  |  |  |
| Ulcerative C |  |  |  |  |  |  |  |  |  |  |  |  |
|  | | | | | | | | | | | | |
| Ankylosing S |  |  |  |  |  |  |  |  |  |  |  |  |
| Psoriatic A |  |  |  |  |  |  |  |  |  |  |  |  |
| Rheumatoid |  |  |  |  |  |  |  |  |  |  |  |  |
|  | | | | | | | | | | | | |
| Multiple S |  |  |  |  |  |  |  |  |  |  |  |  |
| Myasthenia |  |  |  |  |  |  |  |  |  |  |  |  |
|  | Inverse association with HR less than 1 and confidence interval not crossing null | | | | | | | | | | | |
|  | No association HR less than 1 and confidence interval crossing null | | | | | | | | | | | |
|  | Association suggestive of adverse effect but no significant association as confidence interval crossing null | | | | | | | | | | | |
|  | Clear association with HR greater than 1 and confidence interval not crossing null | | | | | | | | | | | |
|  | Significant association with HR above 2 and confidence intervals on the right side | | | | | | | | | | | |

# Table 18 Raw adjusted and Benjamini–Yekutieli–corrected p-values for associations between autoimmune diseases and pregnancy complications.

| Exposures | Outcome | P value  Before correction | P value  After correction | Significant  After Correction |
| --- | --- | --- | --- | --- |
| Any AI | Hyperemesis Gravidarum | 0.001 | 0.014 | Yes |
| Sjogren's syndrome | Hyperemesis Gravidarum | 0.528 | 1.000 | Non-significant before or after correction |
| SLE | Hyperemesis Gravidarum | 0.019 | 0.171 | Non-significant before or after correction |
| Systemic sclerosis | Hyperemesis Gravidarum | 0.017 | 0.171 | Non-significant before or after correction |
| Alopecia areata | Hyperemesis Gravidarum | 0.001 | 0.014 | Yes |
| Psoriasis | Hyperemesis Gravidarum | 0.036 | 0.288 | No |
| Vitiligo | Hyperemesis Gravidarum | 0.012 | 0.144 | No |
| Addison's disease | Hyperemesis Gravidarum | 0.001 | 0.014 | Yes |
| Grave's disease | Hyperemesis Gravidarum | 0.001 | 0.014 | Yes |
| Hashimoto's thyroiditis | Hyperemesis Gravidarum | 0.053 | 0.298 | Non-significant before or after correction |
| T1DM | Hyperemesis Gravidarum | 0.001 | 0.014 | Yes |
| Coeliac disease | Hyperemesis Gravidarum | 0.047 | 0.298 | No |
| IBD | Hyperemesis Gravidarum | 0.152 | 0.729 | Non-significant before or after correction |
| Crohn'sdisease | Hyperemesis Gravidarum | 0.058 | 0.298 | Non-significant before or after correction |
| Ulcerative colitis | Hyperemesis Gravidarum | 0.22 | 0.989 | Non-significant before or after correction |
| Ankyloising spondylitis | Hyperemesis Gravidarum | 0.745 | 1.000 | Non-significant before or after correction |
| Psoriatic arthritis | Hyperemesis Gravidarum | 0.96 | 1.000 | Non-significant before or after correction |
| Rheumatoid arthritis | Hyperemesis Gravidarum | 0.911 | 1.000 | Non-significant before or after correction |
| Multiple sclerosis | Hyperemesis Gravidarum | 0.055 | 0.298 | Non-significant before or after correction |
| Myasthenia gravis | Hyperemesis Gravidarum | 0.045 | 0.298 | Non-significant before or after correction |
| Any AI | Ectopic Pregnancy | 0.001 | 0.024 | Yes |
| Sjogren's syndrome | Ectopic Pregnancy | 0.718 | 1.000 | Non-significant before or after correction |
| SLE | Ectopic Pregnancy | 0.663 | 1.000 | Non-significant before or after correction |
| Systemic sclerosis | Ectopic Pregnancy | 0.24 | 1.000 | Non-significant before or after correction |
| Alopecia areata | Ectopic Pregnancy | 0.119 | 1.000 | Non-significant before or after correction |
| Psoriasis | Ectopic Pregnancy | 0.013 | 0.187 | No |
| Vitiligo | Ectopic Pregnancy | 0.525 | 1.000 | Non-significant before or after correction |
| Addison's disease | Ectopic Pregnancy | 0.646 | 1.000 | Non-significant before or after correction |
| Grave's disease | Ectopic Pregnancy | 0.694 | 1.000 | Non-significant before or after correction |
| Hashimoto's thyroiditis | Ectopic Pregnancy | 0.237 | 1.000 | Non-significant before or after correction |
| T1DM | Ectopic Pregnancy | 0.001 | 0.024 | Yes |
| Coeliac disease | Ectopic Pregnancy | 0.718 | 1.000 | Non-significant before or after correction |
| IBD | Ectopic Pregnancy | 0.026 | 0.312 | No |
| Crohn'sdisease | Ectopic Pregnancy | 0.002 | 0.036 | Yes |
| Ulcerative colitis | Ectopic Pregnancy | 0.455 | 1.000 | Non-significant before or after correction |
| Ankyloising spondylitis | Ectopic Pregnancy | 0.641 | 1.000 | Non-significant before or after correction |
| Psoriatic arthritis | Ectopic Pregnancy | 0.568 | 1.000 | Non-significant before or after correction |
| Rheumatoid arthritis | Ectopic Pregnancy | 0.001 | 0.024 | Yes |
| Multiple sclerosis | Ectopic Pregnancy | 0.723 | 1.000 | Non-significant before or after correction |
| Myasthenia gravis | Ectopic Pregnancy | 0.33 | 1.000 | Non-significant before or after correction |
| Any AI | Miscarriage | 0.001 | 0.024 | Yes |
| Sjogren's syndrome | Miscarriage | 0.007 | 0.101 | No |
| SLE | Miscarriage | 0.739 | 1.000 | Non-significant before or after correction |
| Systemic sclerosis | Miscarriage | 0.215 | 1.000 | Non-significant before or after correction |
| Alopecia areata | Miscarriage | 0.034 | 0.400 | No |
| Psoriasis | Miscarriage | 0.001 | 0.024 | Yes |
| Vitiligo | Miscarriage | 0.05 | 0.400 | No |
| Addison's disease | Miscarriage | 0.15 | 1.000 | Non-significant before or after correction |
| Grave's disease | Miscarriage | 0.854 | 0.036 | No |
| Hashimoto's thyroiditis | Miscarriage | 0.198 | 1.000 | Non-significant before or after correction |
| T1DM | Miscarriage | 0.698 | 0.036 | No |
| Coeliac disease | Miscarriage | 0.935 | 1.000 | Non-significant before or after correction |
| IBD | Miscarriage | 0.002 | 0.036 | Non-significant before or after correction |
| Crohn'sdisease | Miscarriage | 0.582 | 1.000 | Non-significant before or after correction |
| Ulcerative colitis | Miscarriage | 0.001 | 0.024 | Non-significant before or after correction |
| Ankyloising spondylitis | Miscarriage | 0.044 | 0.400 | No |
| Psoriatic arthritis | Miscarriage | 0.286 | 1.000 | Non-significant before or after correction |
| Rheumatoid arthritis | Miscarriage | 0.528 | 1.000 | Non-significant before or after correction |
| Multiple sclerosis | Miscarriage | 0.965 | 1.000 | Non-significant before or after correction |
| Myasthenia gravis | Miscarriage | 0.047 | 0.400 | No |
| Any AI | Gestational hypertension | 0.001 | 0.014 | Yes |
| Sjogren's syndrome | Gestational hypertension | 0.178 | 0.982 | Non-significant before or after correction |
| SLE | Gestational hypertension | 0.001 | 0.014 | Yes |
| Systemic sclerosis | Gestational hypertension | 0.019 | 0.152 | No |
| Alopecia areata | Gestational hypertension | 0.054 | 0.389 | No |
| Psoriasis | Gestational hypertension | 0.001 | 0.014 | Yes |
| Vitiligo | Gestational hypertension | 0.011 | 0.099 | No |
| Addison's disease | Gestational hypertension | 0.004 | 0.048 | Yes |
| Grave's disease | Gestational hypertension | 0.001 | 0.014 | Yes |
| Hashimoto's thyroiditis | Gestational hypertension | 0.191 | 0.982 | No |
| T1DM | Gestational hypertension | 0.001 | 0.014 | Yes |
| Coeliac disease | Gestational hypertension | 0.874 | 1.000 | Non-significant before or after correction |
| IBD | Gestational hypertension | 0.746 | 1.000 | Non-significant before or after correction |
| Crohn'sdisease | Gestational hypertension | 0.077 | 0.462 | Non-significant before or after correction |
| Ulcerative colitis | Gestational hypertension | 0.259 | 1.000 | Non-significant before or after correction |
| Ankyloising spondylitis | Gestational hypertension | 0.685 | 1.000 | Non-significant before or after correction |
| Psoriatic arthritis | Gestational hypertension | 0.851 | 1.000 | Non-significant before or after correction |
| Rheumatoid arthritis | Gestational hypertension | 0.068 | 0.445 | Non-significant before or after correction |
| Multiple sclerosis | Gestational hypertension | 0.006 | 0.062 | Yes |
| Myasthenia gravis | Gestational hypertension | 0.947 | 1.000 | Non-significant before or after correction |
| Any AI | Pre-Eclampsia/Eclampsia | 0.001 | 0.036 | Non-significant before or after correction |
| Sjogren's syndrome | Pre-Eclampsia/Eclampsia | 0.158 | 0.812 | Non-significant before or after correction |
| SLE | Pre-Eclampsia/Eclampsia | 0.002 | 0.048 | Yes |
| Systemic sclerosis | Pre-Eclampsia/Eclampsia | 0.004 | 0.072 | Yes |
| Alopecia areata | Pre-Eclampsia/Eclampsia | 0.085 | 0.510 | Non-significant before or after correction |
| Psoriasis | Pre-Eclampsia/Eclampsia | 0.011 | 0.108 | No |
| Vitiligo | Pre-Eclampsia/Eclampsia | 0.112 | 0.620 | Non-significant before or after correction |
| Addison's disease | Pre-Eclampsia/Eclampsia | 0.015 | 0.120 | No |
| Grave's disease | Pre-Eclampsia/Eclampsia | 0.012 | 0.108 | No |
| Hashimoto's thyroiditis | Pre-Eclampsia/Eclampsia | 0.47 | 1.000 | Non-significant before or after correction |
| T1DM | Pre-Eclampsia/Eclampsia | 0.001 | 0.036 | Yes |
| Coeliac disease | Pre-Eclampsia/Eclampsia | 0.608 | 1.000 | Non-significant before or after correction |
| IBD | Pre-Eclampsia/Eclampsia | 0.056 | 0.366 | No |
| Crohn'sdisease | Pre-Eclampsia/Eclampsia | 0.009 | 0.108 | Yes |
| Ulcerative colitis | Pre-Eclampsia/Eclampsia | 0.768 | 1.000 | Non-significant before or after correction |
| Ankyloising spondylitis | Pre-Eclampsia/Eclampsia | 0.549 | 1.000 | Non-significant before or after correction |
| Psoriatic arthritis | Pre-Eclampsia/Eclampsia | 0.813 | 1.000 | Non-significant before or after correction |
| Rheumatoid arthritis | Pre-Eclampsia/Eclampsia | 0.025 | 0.180 | Non-significant before or after correction |
| Multiple sclerosis | Pre-Eclampsia/Eclampsia | 0.009 | 0.108 | Yes |
| Myasthenia gravis | Pre-Eclampsia/Eclampsia | 0.625 | 1.000 | Non-significant before or after correction |
| Any AI | GDM | 0.001 | 0.017 | No |
| Sjogren's syndrome | GDM | 0.209 | 0.939 | Non-significant before or after correction |
| SLE | GDM | 0.106 | 0.618 | Non-significant before or after correction |
| Systemic sclerosis | GDM | 0.076 | 0.618 | Non-significant before or after correction |
| Alopecia areata | GDM | 0.09 | 0.618 | Non-significant before or after correction |
| Psoriasis | GDM | 0.001 | 0.017 | Yes |
| Vitiligo | GDM | 0.155 | 0.804 | Non-significant before or after correction |
| Addison's disease | GDM | 0.101 | 0.618 | Non-significant before or after correction |
| Grave's disease | GDM | 0.001 | 0.017 | Yes |
| Hashimoto's thyroiditis | GDM | 0.071 | 0.618 | Non-significant before or after correction |
| Coeliac disease | GDM | 0.975 | 1.000 | Non-significant before or after correction |
| IBD | GDM | 0.01 | 0.135 | Non-significant before or after correction |
| Crohn'sdisease | GDM | 0.001 | 0.017 | Non-significant before or after correction |
| Ulcerative colitis | GDM | 0.954 | 1.000 | Non-significant before or after correction |
| Ankyloising spondylitis | GDM | 0.855 | 1.000 | Non-significant before or after correction |
| Psoriatic arthritis | GDM | 0.016 | 0.180 | Non-significant before or after correction |
| Rheumatoid arthritis | GDM | 0.18 | 0.867 | Non-significant before or after correction |
| Multiple sclerosis | GDM | 0.57 | 1.000 | Non-significant before or after correction |
| Myasthenia gravis | GDM | 0.11 | 0.618 | Non-significant before or after correction |
| Any AI | Perinatal anxiety | 0.001 | 0.009 | No |
| Sjogren's syndrome | Perinatal anxiety | 0.065 | 0.360 | Non-significant before or after correction |
| SLE | Perinatal anxiety | 0.001 | 0.009 | Yes |
| Systemic sclerosis | Perinatal anxiety | 0.665 | 1.000 | Non-significant before or after correction |
| Alopecia areata | Perinatal anxiety | 0.173 | 0.835 | Non-significant before or after correction |
| Psoriasis | Perinatal anxiety | 0.001 | 0.009 | Yes |
| Vitiligo | Perinatal anxiety | 0.232 | 1.000 | Non-significant before or after correction |
| Addison's disease | Perinatal anxiety | 0.441 | 1.000 | Non-significant before or after correction |
| Grave's disease | Perinatal anxiety | 0.001 | 0.009 | Yes |
| Hashimoto's thyroiditis | Perinatal anxiety | 0.174 | 0.835 | Non-significant before or after correction |
| T1DM | Perinatal anxiety | 0.025 | 0.150 | No |
| Coeliac disease | Perinatal anxiety | 0.001 | 0.009 | Yes |
| IBD | Perinatal anxiety | 0.001 | 0.009 | Yes |
| Crohn'sdisease | Perinatal anxiety | 0.017 | 0.111 | No |
| Ulcerative colitis | Perinatal anxiety | 0.003 | 0.024 | No |
| Ankyloising spondylitis | Perinatal anxiety | 0.004 | 0.029 | No |
| Psoriatic arthritis | Perinatal anxiety | 0.315 | 1.000 | Non-significant before or after correction |
| Rheumatoid arthritis | Perinatal anxiety | 0.295 | 1.000 | Non-significant before or after correction |
| Multiple sclerosis | Perinatal anxiety | 0.001 | 0.009 | yes |
| Myasthenia gravis | Perinatal anxiety | 0.001 | 0.009 | yes |
| Any AI | Perinatal depression |  | 0.007 | No |
| Sjogren's syndrome | Perinatal depression | 0.042 | 0.216 | No |
| SLE | Perinatal depression | 0.001 | 0.007 | Yes |
| Systemic sclerosis | Perinatal depression | 0.112 | 0.504 | Non-significant before or after correction |
| Alopecia areata | Perinatal depression | 0.346 | 1.000 | No |
| Psoriasis | Perinatal depression | 0.001 | 0.007 | Yes |
| Vitiligo | Perinatal depression | 0.853 | 1.000 | Non-significant before or after correction |
| Addison's disease | Perinatal depression | 0.011 | 0.061 | Yes |
| Grave's disease | Perinatal depression | 0.001 | 0.007 | Yes |
| Hashimoto's thyroiditis | Perinatal depression | 0.083 | 0.398 | No |
| T1DM | Perinatal depression | 0.001 | 0.007 | Yes |
| Coeliac disease | Perinatal depression | 0.011 | 0.061 | No |
| IBD | Perinatal depression | 0.001 | 0.007 | Yes |
| Crohn'sdisease | Perinatal depression | 0.001 | 0.007 | No |
| Ulcerative colitis | Perinatal depression | 0.001 | 0.007 | No |
| Ankyloising spondylitis | Perinatal depression | 0.481 | 1.000 | Non-significant before or after correction |
| Psoriatic arthritis | Perinatal depression | 0.825 | 1.000 | Non-significant before or after correction |
| Rheumatoid arthritis | Perinatal depression | 0.005 | 0.033 | Yes |
| Multiple sclerosis | Perinatal depression | 0.001 | 0.007 | Yes |
| Myasthenia gravis | Perinatal depression | 0.001 | 0.007 | Yes |
| Any AI | Preterm Birth | 0.001 | 0.006 | Yes |
| Sjogren's syndrome | Preterm Birth | 0.125 | 0.529 | Non-significant before or after correction |
| SLE | Preterm Birth | 0.001 | 0.006 | Yes |
| Systemic sclerosis | Preterm Birth | 0.001 | 0.006 | Yes |
| Alopecia areata | Preterm Birth | 0.001 | 0.006 | Yes |
| Psoriasis | Preterm Birth | 0.03 | 0.144 | Yes |
| Vitiligo | Preterm Birth | 0.94 | 1.000 | Non-significant before or after correction |
| Addison's disease | Preterm Birth | 0.001 | 0.006 | Yes |
| Grave's disease | Preterm Birth | 0.001 | 0.006 | Yes |
| Hashimoto's thyroiditis | Preterm Birth | 0.729 | 1.000 | Non-significant before or after correction |
| T1DM | Preterm Birth | 0.001 | 0.006 | Yes |
| Coeliac disease | Preterm Birth | 0.001 | 0.006 | Yes |
| IBD | Preterm Birth | 0.001 | 0.006 | Yes |
| Crohn'sdisease | Preterm Birth | 0.001 | 0.006 | Yes |
| Ulcerative colitis | Preterm Birth | 0.001 | 0.006 | Yes |
| Ankyloising spondylitis | Preterm Birth | 0.722 | 1.000 | Non-significant before or after correction |
| Psoriatic arthritis | Preterm Birth | 0.003 | 0.017 | Yes |
| Rheumatoid arthritis | Preterm Birth | 0.001 | 0.006 | Yes |
| Multiple sclerosis | Preterm Birth | 0.11 | 0.495 | Non-significant before or after correction |
| Myasthenia gravis | Preterm Birth | 0.015 | 0.077 | Yes |
| Any AI | Caesarean Birth | 0.001 | 0.006 | Yes |
| Sjogren's syndrome | Caesarean Birth | 0.063 | 0.302 | Non-significant before or after correction |
| SLE | Caesarean Birth | 0.001 | 0.006 | Yes |
| Systemic sclerosis | Caesarean Birth | 0.354 | 1.000 | Non-significant before or after correction |
| Alopecia areata | Caesarean Birth | 0.431 | 1.000 | Non-significant before or after correction |
| Psoriasis | Caesarean Birth | 0.001 | 0.006 | Non-significant before or after correction |
| Vitiligo | Caesarean Birth | 0.696 | 1.000 | Non-significant before or after correction |
| Addison's disease | Caesarean Birth | 0.001 | 0.006 | Yes |
| Grave's disease | Caesarean Birth | 0.001 | 0.006 | Yes |
| Hashimoto's thyroiditis | Caesarean Birth | 0.211 | 0.949 | Non-significant before or after correction |
| T1DM | Caesarean Birth | 0.001 | 0.006 | Yes |
| Coeliac disease | Caesarean Birth | 0.001 | 0.006 | Yes |
| IBD | Caesarean Birth | 0.001 | 0.006 | Yes |
| Crohn’s disease | Caesarean Birth | 0.001 | 0.006 | Yes |
| Ulcerative colitis | Caesarean Birth | 0.001 | 0.006 | Yes |
| Ankylosing spondylitis | Caesarean Birth | 0.001 | 0.006 | Yes |
| Psoriatic arthritis | Caesarean Birth | 0.001 | 0.006 | Yes |
| Rheumatoid arthritis | Caesarean Birth | 0.001 | 0.006 | Yes |
| Multiple sclerosis | Caesarean Birth | 0.042 | 0.216 | Yes |
| Myasthenia gravis | Caesarean Birth | 0.246 | 1.000 | Non-significant before or after correction |
| Any AI | Stillbirth | 0.001 | 0.031 | No |
| Sjogren's syndrome | Stillbirth | 0.847 | 1.000 | Non-significant before or after correction |
| SLE | Stillbirth | 0.029 | 0.608 | No |
| Alopecia areata | Stillbirth | 0.332 | 1.000 | Non-significant before or after correction |
| Psoriasis | Stillbirth | 0.613 | 1.000 | Non-significant before or after correction |
| Vitiligo | Stillbirth | 0.83 | 1.000 | Non-significant before or after correction |
| Grave's disease | Stillbirth | 0.534 | 1.000 | Non-significant before or after correction |
| Hashimoto's thyroiditis | Stillbirth | 0.161 | 1.000 | Non-significant before or after correction |
| T1DM | Stillbirth | 0.001 | 0.031 | No |
| Coeliac disease | Stillbirth | 0.237 | 1.000 | Non-significant before or after correction |
| IBD | Stillbirth | 0.118 | 1.000 | Non-significant before or after correction |
| Crohn’s disease | Stillbirth | 0.162 | 1.000 | Non-significant before or after correction |
| Ulcerative colitis | Stillbirth | 0.261 | 1.000 | Non-significant before or after correction |
| Ankylosing spondylitis | Stillbirth | 0.886 | 1.000 | Non-significant before or after correction |
| Psoriatic arthritis | Stillbirth | 0.897 | 1.000 | Non-significant before or after correction |
| Rheumatoid arthritis | Stillbirth | 0.438 | 1.000 | Non-significant before or after correction |
| Multiple sclerosis | Stillbirth | 0.576 | 1.000 | Non-significant before or after correction |
| Myasthenia gravis | Stillbirth | 0.449 | 1.000 | Non-significant before or after correction |
| Any AI | Small for Gestational age | 0.001 | 0.024 | No |
| Sjogren's syndrome | Small for Gestational age | 0.673 | 1.000 | Non-significant before or after correction |
| SLE | Small for Gestational age | 0.001 | 0.024 | No |
| Systemic sclerosis | Small for Gestational age | 0.394 | 1.000 | Non-significant before or after correction |
| Alopecia areata | Small for Gestational age | 0.355 | 1.000 | No |
| Psoriasis | Small for Gestational age | 0.634 | 1.000 | No |
| Vitiligo | Small for Gestational age | 0.881 | 1.000 | Non-significant before or after correction |
| Addison's disease | Small for Gestational age | 0.486 | 1.000 | No |
| Grave's disease | Small for Gestational age | 0.666 | 1.000 | Non-significant before or after correction |
| Hashimoto's thyroiditis | Small for Gestational age | 0.023 | 0.331 | Non-significant before or after correction |
| T1DM | Small for Gestational age | 0.001 | 0.024 | Non-significant before or after correction |
| Coeliac disease | Small for Gestational age | 0.596 | 1.000 | Non-significant before or after correction |
| IBD | Small for Gestational age | 0.192 | 1.000 | Non-significant before or after correction |
| Crohn’s disease | Small for Gestational age | 0.817 | 1.000 | Non-significant before or after correction |
| Ulcerative colitis | Small for Gestational age | 0.273 | 1.000 | Non-significant before or after correction |
| Ankylosing spondylitis | Small for Gestational age | 0.311 | 1.000 | Non-significant before or after correction |
| Psoriatic arthritis | Small for Gestational age | 0.627 | 1.000 | Non-significant before or after correction |
| Rheumatoid arthritis | Small for Gestational age | 0.061 | 0.732 | Non-significant before or after correction |
| Multiple sclerosis | Small for Gestational age | 0.002 | 0.036 | Non-significant before or after correction |
| Myasthenia gravis | Small for Gestational age | 0.141 | 1.000 | Non-significant before or after correction |
|  | Statistically significant positive association | | |  |
|  | Statistically significant inverse association | | |  |
